# Loss of ELF2 drives topotecan resistance in retinoblastoma revealed by genome-wide CRISPR-Cas9 screening

**DOI:** 10.1038/s41419-025-08335-z

**Published:** 2025-12-23

**Authors:** Jingyi Jiang, Zihua Jiang, Qian Luo, Xi Chen, Jiejie Zhuang, Jiaxin Chen, Qingqing Mu, Jin Qiu, Yan Li, Shuxia Chen, Ping Zhang, Keming Yu, Shuilian Chen, Guei-Sheung Liu, Jing Zhuang

**Affiliations:** 1 State Key Laboratory of Ophthalmology, Zhongshan Ophthalmic Center, Sun Yat-sen University, Guangdong Provincial Key Laboratory of Ophthalmology and Visual Science, Guangzhou, China; 2 Guangdong Provincial Clinical Research Center for Ocular Diseases, Guangzhou, China; 3 Centre for Eye Research Australia, Royal Victorian Eye and Ear Hospital, East Melbourne, VIC Australia; 4 Ophthalmology, Department of Surgery, University of Melbourne, East Melbourne, VIC Australia; 5 Menzies Institute for Medical Research, University of Tasmania, Hobart, TAS Australia

**Keywords:** Cancer therapeutic resistance, Eye cancer

## Abstract

The topoisomerase I inhibitor topotecan is an effective chemotherapeutic agent for retinoblastoma; however, treatment resistance remains a major clinical challenge, and its mechanisms remain elusive. Using genome-wide CRISPR-Cas9 knockout screening, we identified ELF2 as a key gene involved in topotecan resistance. Here, we show that surviving retinoblastoma cells exposed to topotecan showed progressively decreased ELF2 expression, accompanied by reduced apoptosis. In a mouse xenograft model, ELF2 disruption diminished the antitumor efficacy of topotecan, with ELF2-knockout cells exhibiting reduced topotecan-induced apoptosis. RNA sequencing further revealed that the MT-CYB pathway, associated with ATP synthesis, contributes to ELF2-mediated resistance. Importantly, clinical analysis demonstrated a correlation between ELF2 expression and tumor volume in retinoblastoma patients treated with topotecan. Together, these findings interrogate the mechanisms underlying topotecan resistance in retinoblastoma and suggest ELF2 as a potential therapeutic target to overcome drug resistance.

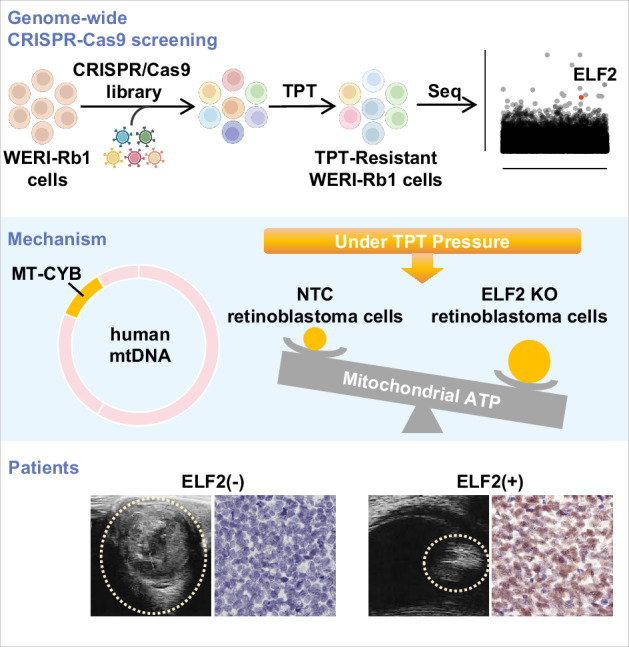

## Introduction

Retinoblastoma is the most common primary intraocular cancer in children, accounting for nearly 4% of paediatric cancers. It is considered fatal when patients present with disseminated and metastatic disease [1–3]. In recent years, treatment modalities have evolved to include enucleation, systemic chemotherapy, intra-arterial chemotherapy (IAC), radiotherapy, and localized treatments such as laser or cryotherapy. Historically, chemotherapy has been used for managing extraocular retinoblastoma since the mid-1990s [4]. The implementation of ophthalmic artery chemosurgery (OAC) has proven effective in treating retinoblastoma cases where systemic chemotherapy shows limited efficacy [5]. In the 2010s, intravitreal chemotherapy (IVi) emerged as a new alternative eye-specific therapeutic option. Additionally, the combination of ocular and intravitreal therapies has become a standard treatment approach in various countries. This evolution has resulted in significant advancements in ocular preservation, greatly diminishing the reliance on external beam radiation therapy [6, 7].

The three most common chemotherapies for intraocular retinoblastoma include melphalan, carboplatin, and topotecan [8]. Topotecan (TPT) is a semi-synthetic derivative of camptothecin, a pentacyclic alkaloid sourced from the *Camptotheca acuminata* tree. It acts primarily in two ways: first, it inhibits the nuclear enzyme topoisomerase I, which causes double-strand breaks during the S-phase of cell replication. It also reduces the activity of the hypoxia-inducible factor [9]. This agent has been approved for the treatment of various malignancies, including cervical cancer, small-cell lung carcinoma, medulloblastoma, neuroblastoma, and rhabdomyosarcoma [9–11]. Preclinical pharmacokinetic studies have shown that topotecan effectively crosses the blood-retina barrier and exhibits excellent vitreous bioavailability [12]. Compared to other chemotherapeutic drugs such as melphalan, topotecan shows higher vitreous concentration levels, a greater vitreous-to-plasma concentration ratio, and improved chemical stability at room temperature. Topotecan has shown effectiveness in both extraocular and refractory intraocular retinoblastoma with minimal ocular and systemic side effects [13–15]. Other studies have also demonstrated that intrathecal administration of topotecan offers a promising treatment approach for retinoblastoma that has spread to the central nervous system [8]. Despite these promising developments, advanced RB frequently advances during multiple chemotherapy cycles and becomes resistant to anticancer treatments, thereby complicating clinical management. Previous studies in various cancers have identified several mechanisms associated with topotecan resistance, including mutations or reduced expression of topoisomerase I and enhanced drug efflux mediated by ATP-binding cassette transporters such as P-glycoprotein, MRP1, and ABCG2 [16–18]. These mechanisms limit intracellular drug accumulation and reduce topotecan cytotoxicity. Prior studies in retinoblastoma have predominantly characterized expression of known drug-resistance proteins using immunohistochemistry and targeted molecular assays [18–22], revealing correlations with treatment outcome but not enabling systematic, genome-wide discovery of novel regulators. Consequently, context-specific or noncanonical mediators of topotecan resistance may have been overlooked, motivating our application of an unbiased, genome-wide CRISPR-Cas9 screening approach [23].

The CRISPR-Cas9 system consists of a Cas9 nuclease and a guide RNA (gRNA), recognized as the pioneering tool for altogether abolishing protein expression by introducing frameshift mutations for specific gene knockout [24, 25]. Essentially, the gRNA library is introduced into a population of cells so that each cell receives only one gRNA. Then, Cas9 proteins cleave the target sequences under the guidance of gRNA. CRISPR-Cas9 knockout cells undergo assays that enable positive or negative selection of the phenotype of interest. The effect of a gene knockout can be measured by comparing the enrichment or depletion of the causative sgRNA relative to its initial abundance in the population [26]. Given its successful application in identifying new drug targets across various types of cancer with genome-wide CRISPR-Cas9 knockout screening [27], we integrated this approach with RNA sequencing to identify key genes and mechanisms that underpin topotecan resistance in retinoblastoma.

In the present study, we identified ELF2, a member of the Ets family of transcription factors, as a novel candidate associated with topotecan resistance using genome-wide CRISPR-Cas9 knockout screening. Our data suggest that knocking out ELF2 causes topotecan resistance by decreasing apoptosis in retinoblastoma both in vitro and in vivo. Furthermore, RNA sequencing analysis of ELF2 knockout cells treated with topotecan revealed that the MT-CYB, which is involved in ATP synthesis, contributes to ELF2-driven topotecan resistance. Thus, this study offers new insights into the mechanisms behind topotecan drug resistance in retinoblastoma, potentially identifying a novel therapeutic target for managing the condition.

## Results

### Genome-wide CRISPR-Cas9 knockout screening identified ELF2 as a critical gene for topotecan resistance

**Fig. 1:**
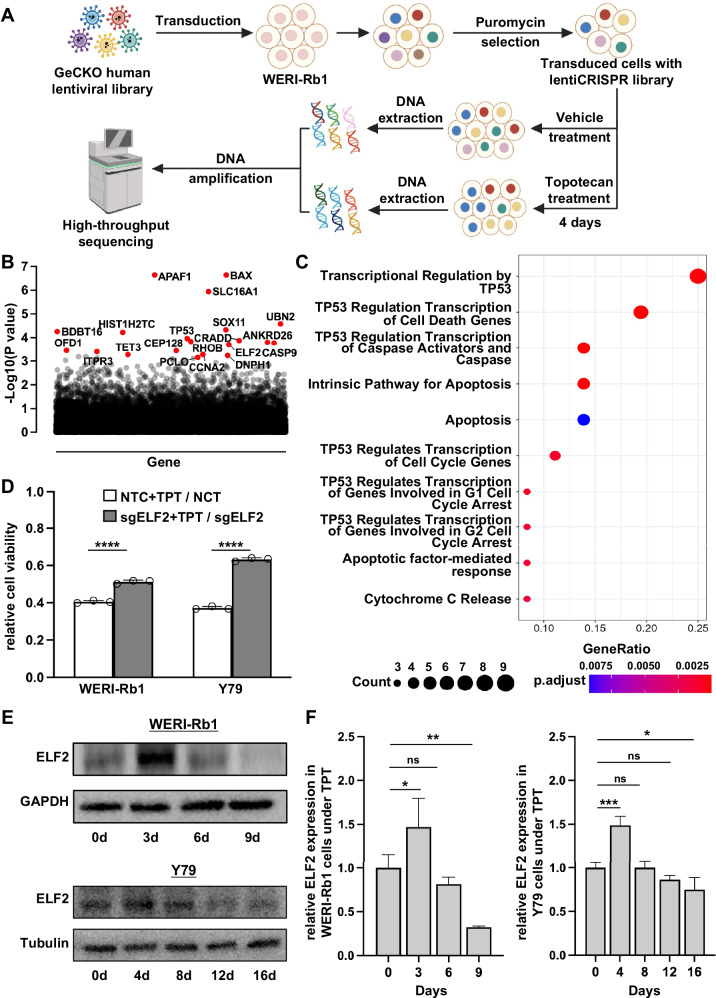
CRISPR library screening identified ELF2 as a driver of topotecan resistance. **A** The schematic diagram illustrates the workflow of genome-wide CRISPR-Cas9 knockout library screening (CRISPR: Clustered Regularly Interspaced Short Palindromic Repeats). **B** The scatter plot depicts the results for topotecan positively selected hits in the CRISPR-Cas9 screening, with the top 20 hits shown in red. **C** KEGG analysis of the top 50 topotecan positively selected hits identified through genome-wide CRISPR-Cas9 knockout screening. **D** Relative cell viability of WERI-Rb1 and Y79 cells following treatment with topotecan or vehicle for 96 hours (*n* = 3). **E**, **F** ELF2 protein expression in WERI-Rb1 and Y79 cells under topotecan treatment (*n* = 3). Data are presented as means ± SD. Statistical analysis was performed using two-tailed Student’s *t* test (**D**) or one-way ANOVA and Tukey’s multiple comparison test (**F**); ****p < 0.0001, ***p < 0.001, **p < 0.01, *p < 0.05.

We conducted a genome-wide screen using a CRISPR-Cas9 knockout library to identify key genes associated with topotecan resistance in a human retinoblastoma cell line. The human GeCKO v2 CRISPR library contains 122,411 unique sgRNAs targeting 19,052 human genes and 1864 miRNAs, which were used to generate a mutant cell pool. We then treated the mutant cell pool with either the vehicle or topotecan for 21 days to enable screening. To impose a strong selection pressure on WERI-Rb1 cells, we applied 100 nM topotecan to the mutant cell pool, a concentration that corresponds to the IC_90_ in this cell line (Fig. 1A**;** Supplementary Fig. 1A). Then, the MAGeCK algorithm was carried out to identify the CRISPR screen hits by calculating the enrichment score indicating the essentiality of a gene (Fig. 1B). We hypothesized that knocking out a gene that drives sensitivity to topotecan would increase the resistance of WERI-Rb1 cells to the drug, preventing cell death or suppression of proliferation caused by the medication. In the presence of topotecan, cells with sgRNAs targeting genes associated with topotecan resistance will be positively enriched in the mutant cell pool, and the identified sgRNAs will then undergo further analysis.

To systematically understand the molecular events associated with topotecan resistance, we performed Gene Ontology (GO) and pathway analyses on the top 50 genes. GO analysis suggested that these genes are involved in apoptosis, DNA damage response, and cell cycle arrest (Supplementary Fig. 2A). Simultaneously, pathway analysis showed that intricate signaling pathways contributed to topotecan sensitivity, including caspase activation, apoptosis, and cell cycle regulation (Fig. 1C). To validate the screening results, the top 20 positively selected genes were individually disrupted using CRISPR-Cas9 in the WERI-Rb1 and Y79 cell lines, both of which are common immortalized human retinoblastoma cell lines. CRISPR-Cas9-mediated gene knockout (KO) and control (non-targeting control; NTC) cells were treated with topotecan or a vehicle for 4 days, followed by a CCK-8 assay to assess cell viability. The changes in survival rates were calculated using the formula: (*S*_(sgRNA+TPT)_/*S*_(sgRNA+veh)_−*S*_(NTC+TPT)_/*S*_(NTC+veh)_)/(*S*_(NTC+TPT)_/*S*_(NTC+veh)_), and were ranked in Supplementary Fig. 2B. Following topotecan treatment, the cell viability of NTC cells was 40.53% in WERI-Rb1 and 37.20% in Y79 cells. Apart from Caspase 9 and BAX, two well-known genes involved in apoptosis, ELF2 knockout achieved the highest cumulative ranking among the 20 genes identified from topotecan-resistant cells. Disruption of the ELF2 gene led to a 26.45% increase in cell viability in WERI-Rb1 cell line and a 69.91% increase in Y79 cell line (Fig. 1D), and was accompanied by a marked elevation in IC_50_ values, from ~30 nM to ~55 nM in WERI-Rb1 cells, and from ~15 nM to ~25 nM in Y79 cells (Supplementary Fig. 1**;** Supplementary Fig. 2C, D). Moreover, we also observed that ELF2 expression responded to topotecan treatment, initially increasing and then significantly declining with continued treatment (Figs. 1E, F; Supplementary Fig. 2E, F).

To better understand the biological significance of ELF2 in retinoblastoma, we further investigated the isoform-specific expression of ELF2. ELF2 encodes two isoforms, ELF2A and ELF2B, which have distinct transcriptional start sites and biological functions [28] (Supplementary Fig. 3A). Notably, ELF2B, a pro-apoptotic isoform [29], was found to be predominantly expressed in both WERI-Rb1 and Y79 cells (Supplementary Fig. 3B), which supports its potential tumor-suppressive role and validates our focus on ELF2 as a topotecan sensitivity-related gene in subsequent analyses. Overall, our results indicate that the loss of ELF2 may contribute to topotecan resistance in retinoblastoma.

### Disruption of the ELF2 gene reduced cellular apoptosis caused by topotecan in vitro

**Fig. 2:**
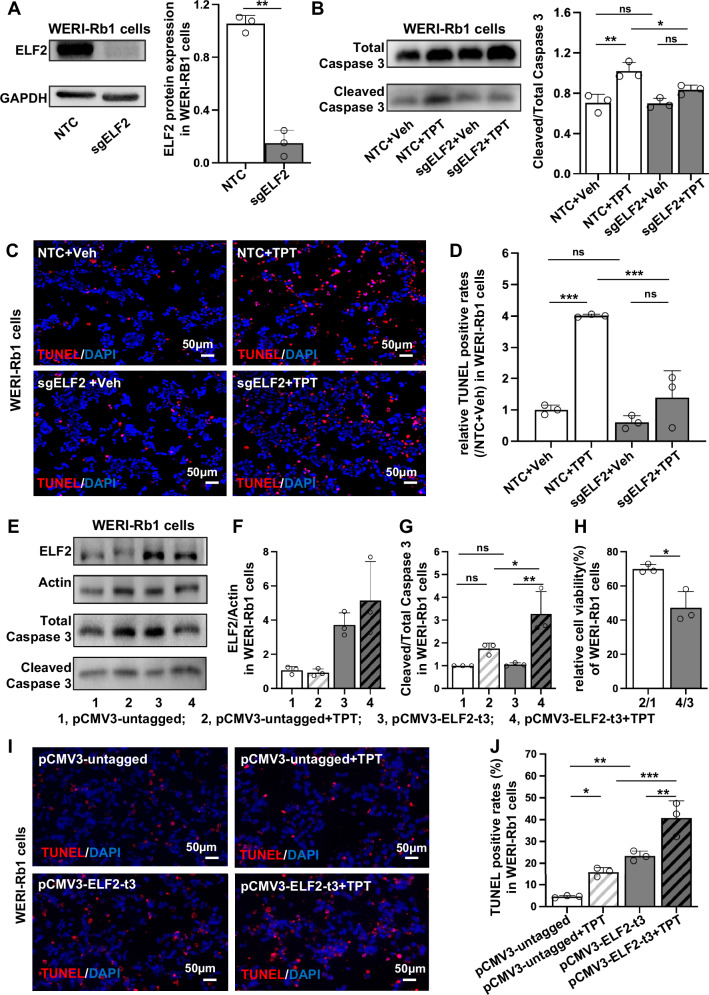
Gene disruption of ELF2 promotes topotecan resistance in WERI-Rb1 cells. **A** A reduction of ELF2 protein expression in ELF2 knockout (KO; sgELF2) cells. **B** Western blot analysis of total caspase-3 and cleaved caspase-3 in topotecan (TPT)-treated ELF2 KO (sgELF2) and non-targeting control (NTC) cells. **C**, **D** Quantitative analysis of apoptotic cells by TUNEL assay in topotecan-treated ELF2 KO (sgELF2) and NTC cells (*n* = 3; scale bar: 50 µm). The relative TUNEL-positive rate was normalized to the mean value of the NT + NaCl group (set as 1.0). **E** Representative western blot image of ELF2 and Caspase-3 proteins in the ELF2-overexpressing (pCMV3-ELF2-t3) and control (pCMV3-untagged) WERI-Rb1 cells. **F**, **G** Quantitative analysis of ELF2 protein, cleaved caspase-3 proteins and total caspase-3 (*n* = 3). Bar groups in **E–G** represent: 1 = pCMV3-untagged, 2 = pCMV3-untagged + TPT, 3 = pCMV3-ELF2-t3, 4 = pCMV3-ELF2-t3 + TPT. **H** Relative cell viability of control and ELF2 overexpressing cells following treatment with topotecan for 72 h (*n* = 3). In **H**, the bars represent fold-change comparisons: 2/1 = (pCMV3-untagged + TPT)/(pCMV3-untagged); 4/3 = (pCMV3-ELF2-t3 + TPT)/(pCMV3-ELF2-t3). **I** and **J** Quantitative analysis of apoptotic cells by TUNEL assay in topotecan-treated ELF2 overexpressing and control cells (*n* = 3; scale bar: 50 µm). Data are presented as means ± SD. Statistical analysis was performed using two-tailed Student’s *t* test (**A**, **H**) or one-way ANOVA and Tukey’s multiple comparison test (**B**, **D**, **F**, **G** and **J**); ****p < 0.0001, ***p < 0.001, **p < 0.01, *p < 0.05.

To further validate the role of ELF2 in topotecan resistance, we constructed ELF2 knockout and overexpression in WERI-Rb1 cells. Western blotting confirmed the reduction of ELF2 protein using CRISPR/Cas9 with the target sgRNA (sgELF2) in WERI-Rb1 cells (Fig. 2A). Caspase-3, known as the executioner caspase, can trigger a rapid feedback loop of caspase activation that ultimately leads to apoptosis. To investigate whether ELF2 knockout attenuates topotecan-induced cell death, a Western blot analysis of caspase-3 cleavage was conducted. While ELF2 knockout alone did not affect caspase activation, it decreased the ratio of cleaved to total caspase-3 in response to drug challenge (Fig. 2B). Consistently, BCL-2/BAX analysis showed that sgELF2 + TPT cells had a higher ratio than NTC + TPT cells, whereas no difference was observed between sgELF2 and NTC under drug-free conditions (Supplementary Fig. 4A, B). Additionally, a TUNEL assay, which labels 3’-OH ends generated by DNA breakage during apoptosis, was conducted to validate apoptotic cells. Likewise, ELF2 knockout cells showed less apoptosis than NTC cells following topotecan exposure (Fig. 2C, D). These results suggest that ELF2 deficiency in WERI-Rb1 cells enhances resistance to topotecan by decreasing apoptosis.

ELF2 overexpression in WERI-Rb1 cells was achieved via transfection using an ELF2 expression vector (pCMV3-ELF2-t3). After 24 h of transfection, the cells were incubated in a culture medium containing topotecan for 72 hours. Western blotting confirmed robust overexpression of the ELF2 protein in transfected cells compared to cells treated with the control vector (pCMV3-untagged; Fig. 2E, F). We observed a significant increase in cell apoptosis, as shown by the ratio of cleaved caspase-3 to total caspase-3 in ELF2-overexpressed cells following topotecan treatment (Fig. 2E, G). Lower relative cell viability in pCMV3-ELF2-t3 cells under 72 h of topotecan treatment indicates that overexpression of ELF2 significantly sensitized retinoblastoma cells to the drug, as shown by CCK8 results (Fig. 2H). In line with caspase-3 activation, the TUNEL assay revealed increased apoptosis in cells overexpressing ELF2 following treatment with topotecan. Interestingly, overexpressing ELF2 in WERI-Rb1 cells caused more apoptosis than in control cells, even without topotecan treatment (Fig. 2I, J). To verify these findings, we further assessed cleaved caspase-3 levels under ELF2 rescue conditions. Western blot analysis revealed that expressed ELF2 restored caspase-3 cleavage to levels similar to control cells, further confirming the role of ELF2 in drug-induced apoptosis (Supplementary Fig. 4C, D). Notably, flow cytometry under topotecan-free conditions showed no significant differences in the cell cycle between ELF2 knockout and controls (Supplementary Fig. 5), ruling out proliferation effects on drug sensitivity. Collectively, these data indicate that ELF2 might be important in regulating cell death in WERI-Rb1 cells following treatment with topotecan.

### Disruption of the ELF2 gene reduces the effectiveness of topotecan in a xenograft mouse model of retinoblastoma in vivo

**Fig. 3:**
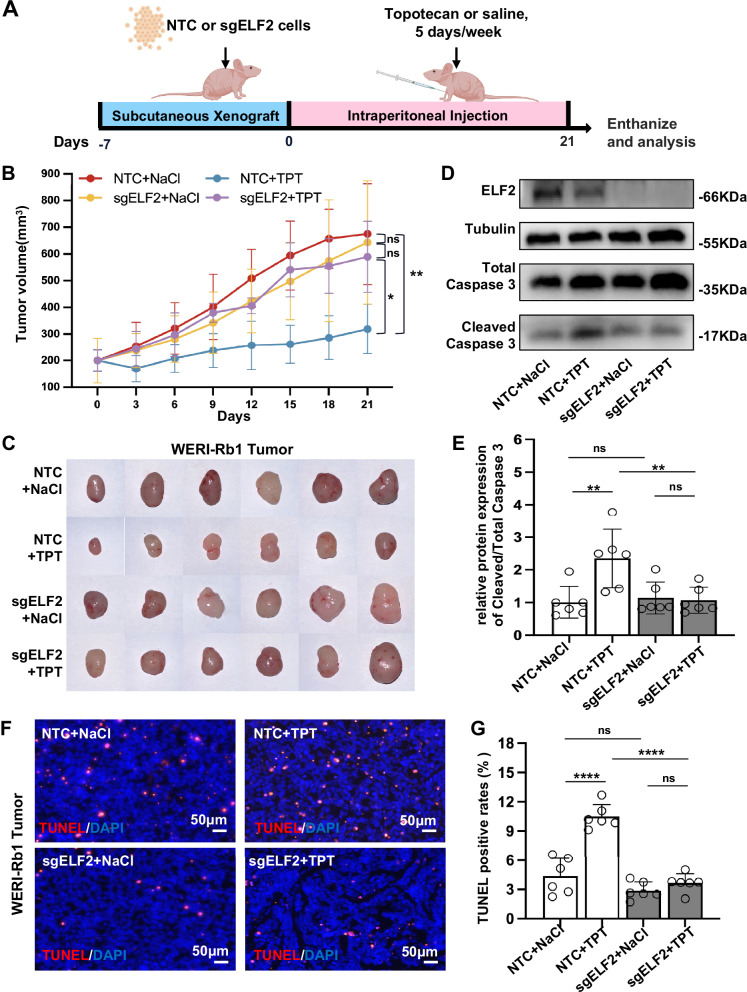
Gene disruption of ELF2 impairs the antitumor effects of topotecan in vivo. **A** Workflow of in vivo mouse xenotransplantation study. Seven days after the transplantation of non-targeting control (NTC) or ELF2 knockout (sgELF2) WERI-Rb1 cells (denoted as day 0), mice were subjected to an intraperitoneal injection of saline or 0.1 mg/kg topotecan (TPT) once daily, Monday through Friday, for 3 consecutive weeks (total 15 doses). **B** Following the initiation of dosing, each mouse was monitored with callipers, and tumor volumes were plotted graphically (*n* = 6). **C** The macroscopic appearance of xenotransplanted tumors 21 days following topotecan treatment. **D** and **E** Representative western blot image and quantitative analysis of ELF2 and Caspase-3 proteins in topotecan-treated ELF2 KO (sgELF2) and NTC tumor tissues (*n* = 6). **F**, **G** Representative image and quantitative analysis of apoptotic cells by TUNEL assay in topotecan-treated ELF2 KO (sgELF2) and NTC tumor tissues (*n* = 6; scale bar: 50 µm). Data are presented as means ± SD. Statistical analysis was performed using one-way ANOVA and Tukey’s multiple comparison test (**B**, **E** and **G**); ****p < 0.0001, **p < 0.01, *p < 0.05.

To evaluate how disrupting the ELF2 gene impacts topotecan resistance in vivo, ELF2 knockout or NTC WERI-Rb1 cells were subcutaneously inoculated into nude mice. When the volume of the tumors reached 200 mm³, the mice were randomly assigned to receive either a daily intraperitoneal injection of topotecan (at a dose of 0.1 mg/kg, 5 days per week) or an equivalent volume of saline (Fig. 3A). Tumor dimensions were measured every three days using callipers, and tumor volume was plotted graphically in Fig. 3B. Volumetric measurements indicated that topotecan led to a significant reduction in tumor size in mice with NTC cells compared to those receiving saline treatment. Conversely, there was no difference in tumor size observed in ELF2 knockout cells, irrespective of whether they were treated with topotecan or saline. The tumor volume was further confirmed by excising the xenotransplanted tumor tissues at 21 days post-treatment (Fig. 3C). The reduction in topotecan-induced cell apoptosis was further observed in the ELF2 knockout tumor tissues compared to NTC tumor tissues via western blotting of caspase-3 and TUNEL assays (Fig. 3D-G). Overall, our results suggest that ELF2 may act as a checkpoint that initiates tumor cell death during chemotherapy; therefore, the loss of ELF2 could lead to topotecan resistance by opposing these cellular responses.

### RNA sequencing revealed that MT-CYB is involved in ELF2-associated topotecan resistance

**Fig. 4:**
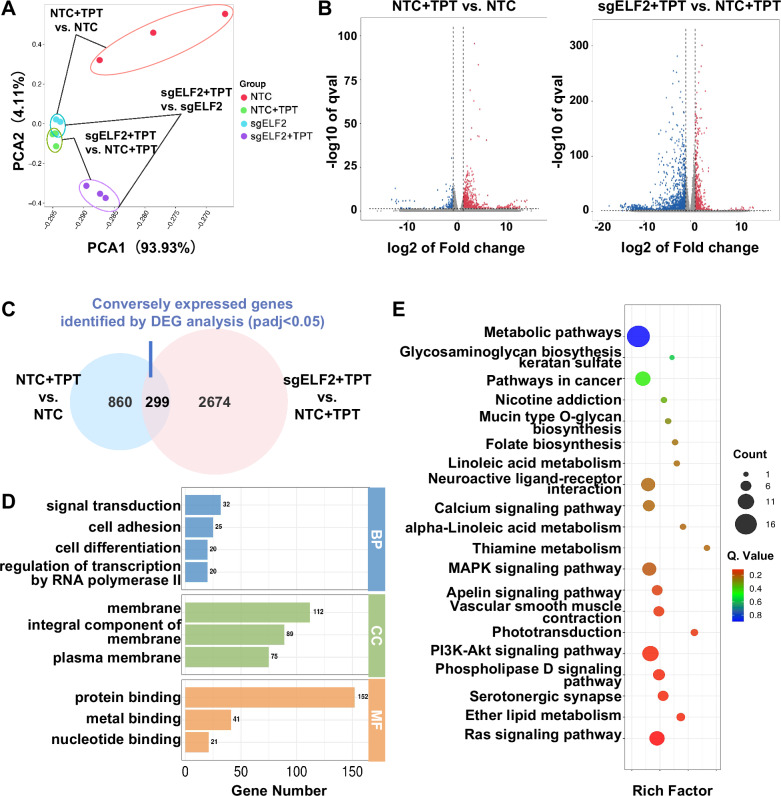
Gene disruption of ELF2 enhances resistance to topotecan through metabolic pathways. **A** Principal component analysis (PCA) of RNA-seq data from the experimental groups (non-targeting control (NTC), ELF2 knockout (KO; sgELF2), topotecan(TPT)-treated NTC, topotecan-treated ELF2 KO WERI-Rb1 cells; *n* = 3). **B** Volcano plots illustrate the genes that were significantly altered in both NTC and ELF2 KO (sgELF2) WERI-Rb1 cells upon stimulation with topotecan. Red and blue dots indicate genes exhibiting a log2|fold change | > 1 and FDR < 0.05. **C** Venn diagram of differentially expressed genes (DEGs) between the two data sets. **D** Gene Ontology and **E** KEGG enrichment analysis of the identified genes from (**C**).

To investigate the transcriptional mechanisms by which ELF2 loss confers topotecan resistance, we generated the transcriptome profile of WERI-Rb1 NTC and sgELF2 cells, both with and without topotecan exposure. Principal component analysis revealed clear segregation between groups, indicating distinct transcriptional changes driven by ELF2 loss and topotecan exposure (Fig. 4A). To identify genes whose transcriptional response to topotecan depends on ELF2, we performed differential expression analyses (padj < 0.05) for two comparisons: NTC + TPT versus NTC, and ELF2 knockout (sgELF2) + TPT versus NTC + TPT. We then selected 299 genes that were regulated in opposite directions between these two comparisons, upregulated by topotecan in control cells but downregulated in ELF2-deficient cells, or vice versa (Fig. 4B, C). To confirm that these genes were not solely affected by ELF2 loss, we further examined their significance in NTC versus sgELF2 and sgELF2 versus sgELF2 + TPT comparisons. Of 299 genes, 143 (47.8%) showed no significant difference at baseline between NTC and sgELF2, and 179 (59.9%) showed no significant change between sgELF2 and sgELF2 + TPT (Supplementary Fig. 6). GO terms were enriched for signal transduction, cell adhesion, membrane components, and protein/metal ion binding, while KEGG analysis highlighted metabolic and Ras signaling pathways (Fig. 4D, E).

**Fig. 5:**
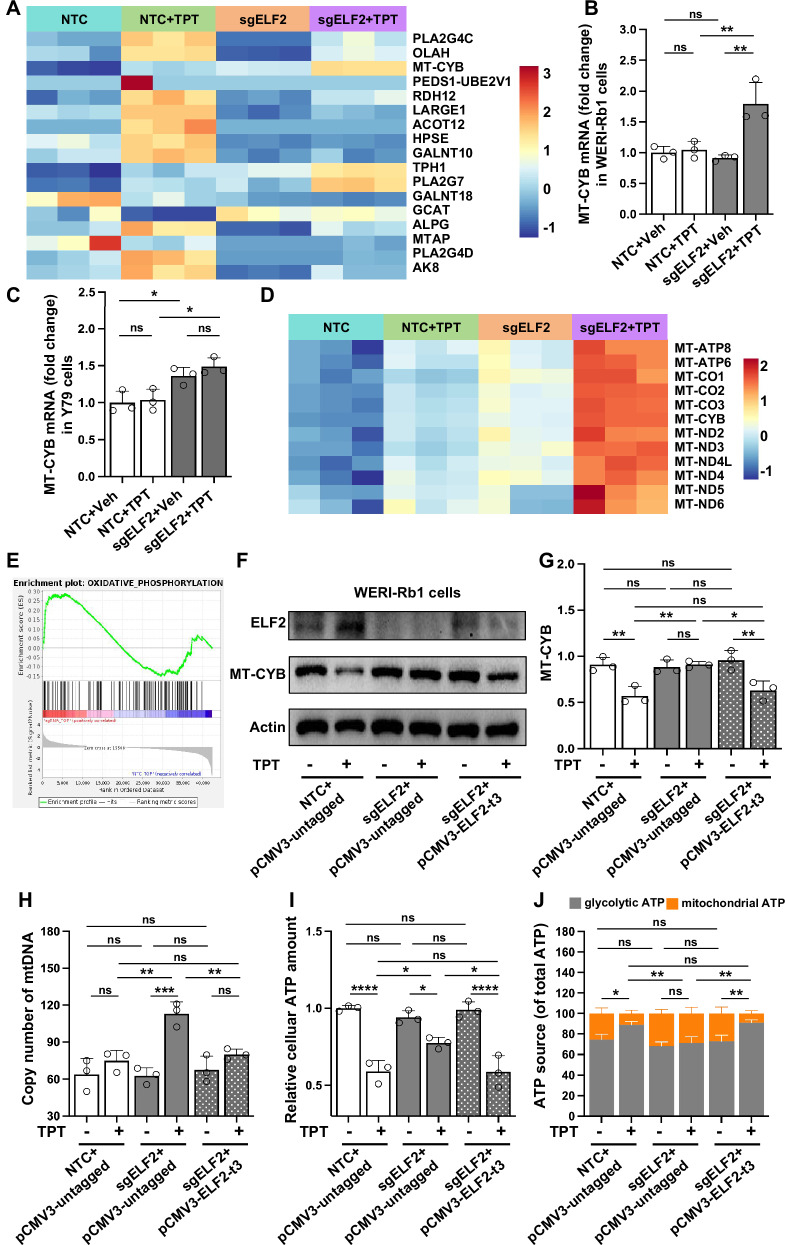
Resistance of ELF2 KO cells to topotecan is related to mitochondrial metabolic alterations. **A** Heatmap illustrating the expression of 17 genes involved in the metabolic pathways identified in Fig. 4E for each sample across various groups. **B** The mRNA levels of MT-CYB in WERI-Rb1 cells across different groups. **C** The mRNA levels of MT-CYB in Y79 cells across different groups. **D** Heatmap illustrating the expression of 12 mtDNA-encoded genes across groups. **E** GSEA enrichment plots of OXIDATIVE_PHOSPHORYLATION pathways comparing sgELF2 + topotecan(TPT) vs. NTC + TPT. **F**, **G** Representative Western blot and quantitative analysis of MT-CYB expression in control (NTC + pCMV3-untagged), ELF2 knockout (sgELF2 + pCMV3-untagged), and ELF2 rescue (sgELF2 + pCMV3-ELF2-t3) cells. **H**, **I** Quantitative analysis of mitochondrial DNA copy number and cellular ATP levels in WERI-Rb1 cells across various groups. **J** The relative contribution of mitochondrial and glycolytic ATP across different groups. Data are presented as means ± SD. Statistical analysis was performed using one-way ANOVA and Tukey’s multiple comparison test (**B**, **C**, **G**, **H**, **I** and **J**); ****p < 0.0001, ***p < 0.001, **p < 0.01, *p < 0.05.

Alterations in metabolism are closely linked to cancer etiology and drug resistance [30, 31]. Therefore, we further investigated metabolism-related genes (Fig. 5A, Supplementary Fig. 7). Among these, MT-CYB emerged as a candidate due to its relatively high expression, consistent regulation across both WERI-Rb1 and Y79 cell lines, and concordance between RNA-seq and qPCR validation (Fig. 5B, C). MT-CYB, encoded by mitochondrial DNA, produces cytochrome b, a core component of the ubiquinol-cytochrome c reductase complex in the mitochondrial respiratory chain [32]. Furthermore, transcriptomic profiling revealed that multiple other mtDNA-encoded genes, including MT-ATP6, MT-ATP8, MT-CO1-CO3, and MT-ND2-ND6, which encode essential subunits of the electron transport chain complexes I, III, IV, and V, were also elevated in topotecan-treated ELF2 knockout cells (Fig. 5D). These coordinated changes suggest a broad activation of oxidative phosphorylation (OXPHOS) in response to ELF2 depletion under drug treatment. qPCR validation of TFAM, ND1, COX1, and ATP6 confirmed these transcriptomic alterations (Supplementary Fig. 8A). In line with these gene-level changes, GSEA analysis comparing sgELF2+TPT versus NTC + TPT revealed the positive enrichment for OXIDATIVE_PHOSPHORYLATION (NES > 0; Fig. 5E) and negative enrichment for AEROBIC_RESPIRATION (NES < 0; Supplementary Fig. 8B), while GSVA heatmaps showed heightened activity in ATP_BIOSYNTHETIC_PROCESS, REGULATION_OF_OXIDATIVE_PHOSPHORYLATION, and related pathways in sgELF2 and especially sgELF2+TPT cells (Supplementary Fig. 8C).

To assess functional consequences of these transcriptomic changes, we examined mitochondrial activity at various levels using updated six-group comparisons (NTC, ELF2 knockout ELF2-rescue ± topotecan). In NTC cells, topotecan markedly reduced MT-CYB protein levels, whereas in ELF2 knockout cells, MT-CYB levels remained largely unchanged after topotecan, and this pattern was abolished by ELF2 rescue (Figs. 5F, G), indicating that ELF2 loss sustains mitochondrial activity under drug pressure. Notably, while MT-CYB mRNA levels remained relatively unchanged in NTC cells following topotecan treatment (Fig. 5B, C), its protein expression decreased significantly (Fig. 5F), aligning with stress-induced post-transcriptional regulation such as translational repression [33] and protein degradation [34]. In contrast, ELF2 knockout cells showed increased MT-CYB mRNA and stable protein levels upon topotecan treatment (Fig. 5B, C, F), suggesting reduced stress-mediated suppression of mitochondrial translation. In terms of mitochondrial content, the copy number of mtDNA, a proxy for mitochondrial biogenesis and function [35], was markedly increased in ELF2 knockout + topotecan cells and reversed upon ELF2 rescue (Fig. 5H). To assess mitochondrial output, we measured total ATP levels and their sources. In our system, ATP measurement revealed no significant differences between groups under baseline conditions; however, upon topotecan exposure, ATP levels declined sharply in NTC cells but were preserved mainly in ELF2 knockout cells, with this effect reversed by ELF2 re-expression (Fig. 5I). To distinguish the source of ATP, we treated cells with oligomycin to inhibit mitochondrial ATP synthesis. The proportion of mitochondrial-derived ATP increased by approximately 150% in ELF2 knockout + topotecan cells compared to NTC + topotecan, and this shift was abolished in the rescue group (Fig. 5J). These findings suggest that ELF2 deficiency enables a metabolic adaptation under chemotherapeutic stress, enhancing OXPHOS-dependent ATP production and potentially supporting cell survival.

Together, these data support the model that ELF2 loss promotes mitochondrial reprogramming and maintains OXPHOS activity under topotecan treatment, contributing to drug resistance. Restoring ELF2 reverses these mitochondrial effects, highlighting its vital role in regulating cellular response to chemotherapy.

### Disruption of the ELF2 gene decreased apoptosis following topotecan treatment in Y79 retinoblastoma

**Fig. 6:**
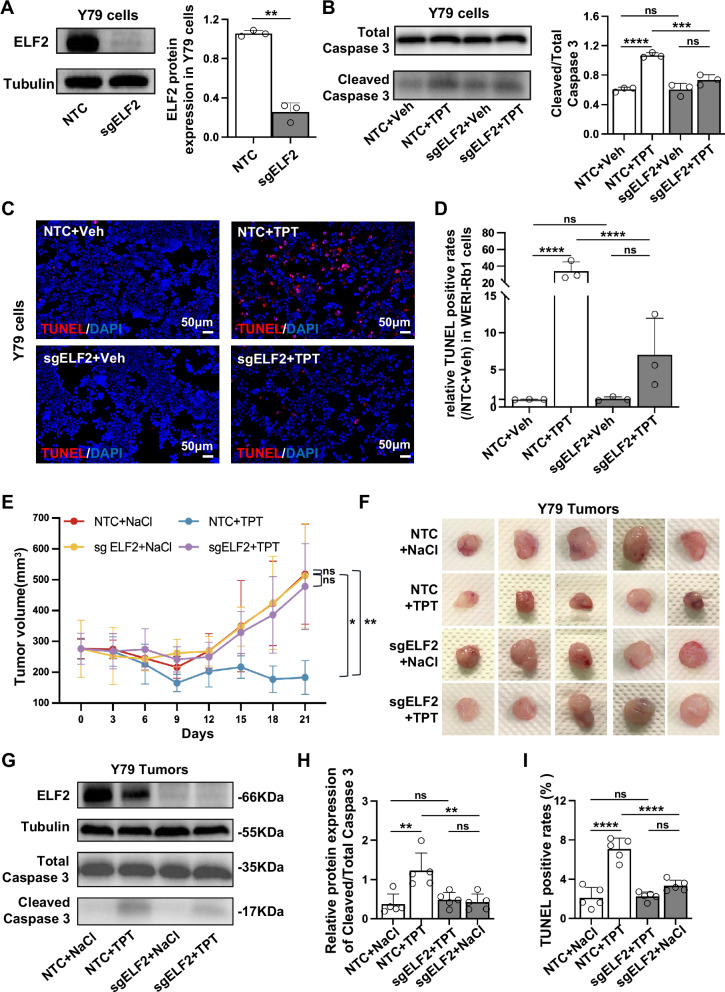
Gene disruption of ELF2 enhances resistance to topotecan in Y79 retinoblastoma cells. **A** A reduction of ELF2 protein expression in ELF2 knockout (KO; sgELF2) cells compared to non-targeting control (NTC) cells. **B** Western blot analysis of total caspase-3 and cleaved caspase-3 in topotecan (TPT)-treated ELF2 KO and NTC cells. **C**, **D** Quantitative analysis of apoptotic cells by TUNEL assay in topotecan-treated ELF2 KO and NTC cells (n = 3; scale bar: 50 µm). The relative TUNEL-positive rate was normalized to the mean value of the NT + NaCl group (set as 1.0). **E** Seven days after the transplantation of NTC or ELF2 KO (sgELF2) cells (denoted as day 0), mice were subjected to an intraperitoneal injection of saline or 0.05 mg/kg topotecan once daily, Monday through Friday, for three consecutive weeks (total 15 doses). Following the initiation of dosing, each mouse was monitored with callipers, and tumor volumes were plotted graphically (*n* = 5). **F** The macroscopic appearance of xenotransplanted tumors 21 days following topotecan treatment. **G** and **H** Representative western blot image and quantitative analysis of ELF2 and Caspase-3 proteins in topotecan-treated ELF2 KO (sgELF2) and NTC tumor tissues (*n* = 5). **I** Quantitative analysis of apoptotic cells by TUNEL assay in topotecan-treated ELF2 KO (sgELF2) and NTC tumor tissues (*n* = 5). Data are presented as means ± SD. Statistical analysis was performed using two-tailed Student’s *t* test (**A**) or one-way ANOVA and Tukey’s multiple comparison test (**B**, **D**, **E**, **H** and **I**); ****p < 0.0001, ***p < 0.001, **p < 0.01, *p < 0.05.

Given that the foundation of our experiment was only based on the WERI-Rb1 cell line, we therefore carried out in vivo and in vitro validations using another retinoblastoma cell line, Y79, to further elucidate the role of ELF2 in retinoblastoma. We first verified the disruption of the ELF2 gene in Y79 cells using the CRISPR/Cas9 system with western blotting (Fig. 6A). After treating Y79 cells with topotecan, we observed a significant decrease in the ratio of cleaved caspase-3 to total caspase-3 in ELF2 knockout cells compared to NTC cells (Fig. 6B). Consistently, BCL-2/BAX analysis showed a higher ratio in sgELF2 + topotecan cells (Supplementary Fig. 9A, B). TUNEL assay also showed that ELF2 knockout cells had less apoptosis than NTC cells following topotecan treatment (Fig. 6C, D). Additionally, similar to WERI-Rb1 cells, the overexpression of the ELF2 gene encourages apoptosis and reduces cell viability in Y79 cells following topotecan treatment (Supplementary Fig. 8C-H). Flow cytometry under topotecan-free conditions revealed no significant cell cycle differences between ELF2 knockout Y79 cells and controls (Supplementary Fig. 10).

Subsequently, a xenotransplant model of Y79 retinoblastoma was employed to elucidate the role of ELF2 in vivo. Volumetric measurements indicated that topotecan treatment led to a significant reduction in tumor size in mice with NTC cells compared to those receiving saline treatment. However, there was no significant difference in the growth of ELF2 knockout tumors between the saline or topotecan treatments (Fig. 6E). The tumor volume was further confirmed by excising the xenotransplanted tumor tissues at 21 days post-treatment (Fig. 6F). The reduction in topotecan-induced cell apoptosis was further observed in ELF2 knockout tumor tissues compared to NTC tumor tissues, as shown by western blotting of caspase-3 and TUNEL assays (Fig. 6G-I**;** Supplementary Fig. 11). Overall, these findings further confirm our hypothesis that the loss of ELF2 could lead to topotecan resistance in retinoblastoma.

### Evaluating the clinical significance of ELF2 in the resistance to topotecan using human clinical retinoblastoma samples

**Fig. 7:**
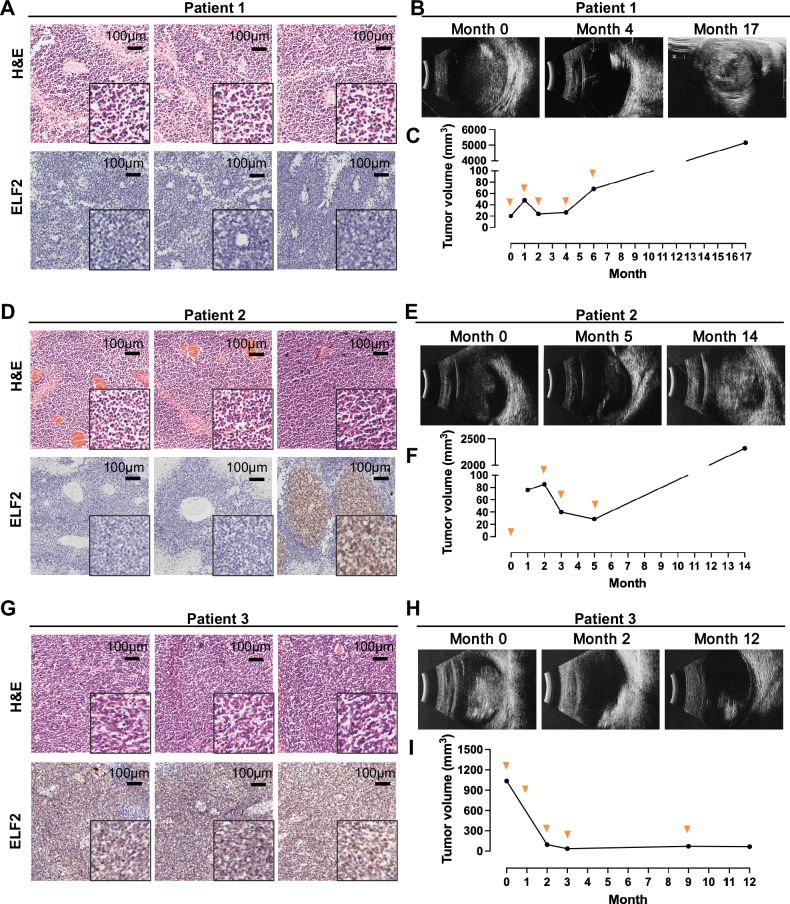
The correlation between ELF2 and the response to topotecan in clinical samples. Representative images of H&E, ELF2 immunohistochemical staining and ultrasound of patient 1 (**A**, **B**), patient 2 (**D**, **E**) and patient 3 (**G**, **H**) (IHC, scale bar: 100 µm). Line graph of tumor volumes of patient 1 (**C**), patient 2 (**F**) and patient 3 (**I**). The orange dots in the line graphs indicate topotecan treatment points.

To further explore the clinical significance of ELF2 in topotecan resistance, we examined its association with topotecan resistance using patient retinoblastoma samples. To evaluate ELF2 expression in retinoblastoma, we collected post-surgical specimens from nine patients who had undergone topotecan treatment and later required globe enucleation. We collected ultrasound imaging and ELF2 immunohistochemical (IHC) staining data to assess the correlation between ELF2 expression and the response to topotecan therapy. Figure 7 highlights three representative patients showing low, moderate, and high ELF2 expression levels in their retinoblastoma tissues. In patient 1, the tumor tissue showed minimal or absent ELF2 protein expression, correlating with a limited response to recurrent topotecan treatments. After five cycles of topotecan therapy, tumor enlargement was observed compared to its initial diagnosis size (Fig. 7A-C). Similarly, patient 4 exhibited a comparable pattern of ELF2 expression and drug response as patient 1 (Supplementary Fig. 12A). In patient 2, a partial absence of ELF2 protein led to a partial response to topotecan, with tumor volume decreasing from approximately 100 mm³ to 20 mm³ (Fig. 7D-F). In contrast, tumor tissue from patient 3 showed high levels of ELF2 expression, correlated with a positive response to topotecan therapy and a notable decrease in tumor size from over 1000 mm³ to less than 100 mm³ after four treatment cycles (Fig. 7G-I). Although globe salvage therapies for patient 3 were successful, enucleation was performed due to familial considerations. Patients 5 to 9 also showed high ELF2 expression levels in their retinoblastomas; however, the loss of follow-up led to disease progression that required subsequent globe enucleation (Supplementary Fig. 12B-F). These observations indicate that the reduction of ELF2 levels contributes to resistance to topotecan and could serve as a reliable biomarker for predicting patient responses to topotecan treatment, while also offering precise guidance for clinical intervention.

## Discussion

Topotecan, a topoisomerase I inhibitor, is commonly used to treat retinoblastoma due to its effective ocular penetration and relatively low systemic toxicity. However, resistance to topotecan remains a significant challenge for long-term treatment success. Previous studies have implicated mechanisms such as enhanced drug efflux [18–21, 36] and altered topoisomerase expression [16–18], but the full range of molecular factors causing topotecan resistance remains only partly understood.

To tackle this challenge, we used a genome-wide CRISPR-Cas9 loss-of-function screen in retinoblastoma cells to identify genes whose knockout confers resistance to topotecan. This approach offers an unbiased and high-throughput way to identify key regulators of drug response, especially those with essential or context-specific roles [27]. Our screen identified ELF2 as a top hit. ELF2, a member of the ETS transcription factor family, exhibits context-dependent roles in cancer. It promotes tumor growth in osteosarcoma, hepatocellular carcinoma, and nasopharyngeal cancer [37–39], but the ELF subfamily, including ELF1 and ELF4, shows anti-proliferative effects in fibrosarcoma and T3M-1 squamous cell carcinoma cells [40]. This duality may be partly explained by the existence of two isoforms, ELF2A and ELF2B, which differ by 12 amino acids [28] and are transcribed from alternative promoters [29] (Supplementary Fig. 3A). Specifically, ELF2B acts as a dominant negative regulator compared to ELF2A, functioning as a tumor suppressor gene that triggers apoptosis. In our study, ELF2B was the primary isoform in both WERI-Rb1 and Y79 cells, with ELF2A expressed at considerably lower levels (Supplementary Fig. 3B).

We also observed a transient increase in ELF2 expression at both mRNA and protein levels upon topotecan treatment, followed by a sustained decline at later time points. This suggests that ELF2 regulation occurs at least partly at the transcriptional level. Prior studies indicate that ELF2 transcription is regulated through alternative promoters, producing isoforms (ELF2A and ELF2B) with differential responses to cellular signals such as hypoxia and angiopoietin-1 via ERK1/2 activation [28, 41]. Post-transcriptionally, microRNAs, including miR-409-3p and miR-188 have been shown to reduce ELF2 mRNA stability or translation efficiency [39, 42]. Although direct evidence on ELF2 protein turnover is lacking, studies on other ETS family members reveal regulation via ubiquitin-proteasome pathways, as seen in SPOP-mediated ERG degradation and hypoxia-induced ETS1 proteolysis [43, 44], suggesting ELF2 may be similarly controlled.

Our data suggest that the downregulation of ELF2 diminished TPT-induced apoptosis and cytotoxicity in both WERI-Rb1 and Y79 retinoblastoma cell lines, both in vitro and in vivo. Functional rescue and overexpression experiments further supported ELF2’s role in topotecan sensitivity: ELF2 re-expression in knockout cells increased caspase-3 activation, while ELF2 overexpression in NTC cells enhanced topotecan-induced apoptosis and reduced viability in both retinoblastoma lines (Fig. 2 and Fig. 6**;** Supplementary Fig. 4). Furthermore, although our patient cohort was small, we observed an inverse correlation between ELF2 expression and tumor burden during topotecan treatment, suggesting potential clinical relevance (Fig. 7). Consistent with our findings, other ELF family members have also been implicated in drug resistance. Notably, the loss of ELF1, a closely related factor, promotes resistance to docetaxel and cisplatin in prostate cancer [45, 46] and cisplatin resistance in non-small cell lung cancer [47], suggesting that dysregulation of ELF-family transcription factors may contribute more broadly to chemoresistance. Interestingly, ELF1 was previously shown to interact with the retinoblastoma (Rb) protein [48], suggesting a regulatory link between Ets factors and Rb signaling. As Rb function is lost in retinoblastoma cells such as WERI-Rb1 and Y79, whether ELF2 acts through Rb-independent mechanisms remains to be explored.

To investigate ELF2’s downstream effects on topotecan sensitivity, transcriptome analysis identified MT-CYB, a mitochondrially encoded core subunit of respiratory complex III, as a key mediator (Fig. 5). MT-CYB was consistently upregulated in ELF2 knockout cells under topotecan across two retinoblastoma cell lines and validated by qPCR. This upregulation also affected other mitochondrial OXPHOS genes, along with increased mitochondrial DNA copy number, higher ATP production, and a shift to mitochondrial ATP dependence, all of which were reversed by ELF2 re-expression. Transcriptional enrichment analyses (GSEA and GSVA) further confirmed increased activity of the OXPHOS pathway in ELF2-deficient cells under topotecan.

Similar OXPHOS-related metabolic adaptations have been reported in various cancers with chemoresistant phenotypes, including leukemia, breast cancer, and pancreatic cancers [49–55]. MT-CYB overexpression has been shown to promote proliferation and invasiveness in bladder cancer [56] and to be associated with poor prognosis in colorectal cancer [57]. Furthermore, Chang et al. associated a MT-CYB genetic variant (rs2853493) and its expression levels with neuroblastoma risk in large pediatric cohorts [58]. These findings collectively underscore the importance of MT-CYB in tumor progression and chemoresistance, supporting the biological and translational relevance of targeting the MT-CYB/OXPHOS axis in retinoblastoma.

Beyond mitochondrial metabolism, KEGG and GO analyses also revealed ELF2-regulated pathways such as PI3K-Akt, Ras signalling, and cell adhesion, indicating broader roles for ELF2 in chemoresistance. Further research is necessary to better understand their role in the ELF2-mediated response to topotecan. To comprehensively map the network of topotecan response regulators, we are conducting negative selection and CRISPRa-based overexpression screens in future studies to uncover additional modulators of TPT response, potentially expanding the therapeutic target landscape.

Taken together, our findings indicate that, under topotecan treatment, ELF2 restrains mitochondrial activation and OXPHOS upregulation, thereby preserving the drug’s cytotoxic efficacy. Loss of ELF2 triggers metabolic changes leading to increased mitochondrial respiration, helping retinoblastoma cells sustain energy and avoid apoptosis. These insights deepen our understanding of how mitochondria contribute to chemoresistance and highlight ELF2 as a potential biomarker and therapeutic target for improving topotecan-based treatment strategies.

## Materials and methods

### Genome-wide CRISPR-Cas9 knockout screening in retinoblastoma

The Human CRISPR Knockout Pooled Library (hGeCKO v2; Addgene #1000000048) was utilized to identify the genes responsible for topotecan resistance in retinoblastoma cells. In brief, we transduced WERI-Rb1 cells with the GeCKO v2 library, which comprises 122,411 unique sgRNA sequences targeting 19,052 human genes and 1864 miRNAs (with six sgRNAs per gene, four sgRNAs per miRNA, and 1000 non-targeting controls) at a low multiplicity of infection (MOI) of approximately 0.3 to ensure effective barcoding of individual cells. Following this transduction process, the modified cells were cultured in a medium containing 2 μg/mL puromycin for 7 days to generate a mutant cell pool. Subsequently, this mutant cell pool was treated with either vehicle (DMSO) or topotecan (100 nM; APExBIO B4982) for 21 days. In vitro drug sensitivity assessment of WERI-Rb1 cells treated with serial dilutions of topotecan for 96 h determined that 100 nM approximates the IC_90_ and was therefore selected to apply sufficient selection pressure on retinoblastoma cells (Supplementary Fig. 1A). A total input of 1 μg DNA per sample was employed for DNA sample preparations. Sequencing libraries were constructed using the NEBNext® Ultra™ DNA Library Prep Kit for Illumina (NEB, USA) and subjected to high-throughput amplicon sequencing conducted by Novogene Technology (Beijing, China). The analysis of CRISPR screen data was performed using MAGeCK (Model-based Analysis of Genome-wide CRISPR/Cas9 Knockout) v0.5.9.3 [59]. Read counts from all samples were first median-normalized. A negative binomial model was used to assess the significance of differences in sgRNA abundance between treatment and control groups. sgRNAs were ranked by their p-values, and gene-level scores were calculated using a modified robust rank aggregation algorithm (α-RRA), which prioritizes genes with consistently enriched or depleted sgRNAs. False discovery rates (FDRs) were then computed for gene-level significance. For Fig. 1B, we plotted -log10(FDR) against the corresponding effect size for each gene, and the top 20 positively selected hits were highlighted in red.

### Cell lines and culture

The human WERI-Rb1 and Y79 cell lines were purchased from American Type Culture Collection (ATCC, Manassas, VA, USA). Subsequently, the cells were cultured in RPMI-1640 medium (Gibco; Thermo Fisher Scientific, Inc) supplemented with 10% fetal bovine serum (FBS; ExCell Bio, FSP500). The culture conditions were maintained at 37 °C in a humidified atmosphere of 5% CO_2_ and 95% air to ensure optimal cell growth. Both cell lines were authenticated by short tandem repeat (STR) profiling in 2020 (Guangzhou Cellcook Biotech Co., Ltd), confirming their identity with reference databases and excluding cross-contamination.

### Xenotransplant mouse model of retinoblastoma

Twenty or twenty-four BALB/c-nu nude mice (4–6 weeks old, both male and female, balanced between groups) were utilized for the xenotransplant model. The animals received subcutaneous injections in the right flank with 5 × 10^6^ transduced WERI-Rb1 or Y79 cells suspended in 0.3 mL of a 1:1 mixture of ice-cold Matrigel. One week post-injection, the animals were assessed for successful tumor transplantation. Upon confirmation of successful tumor establishment (tumor volume > 200 mm³), the mice were randomly assigned to receive either an intraperitoneal injection of topotecan (0.1 mg/kg for tumors derived from WERI-Rb1 and 0.05 mg/kg for those from Y79) or an equivalent volume of saline on weekdays over three weeks. Tumor dimensions were measured every three days using callipers; both the longest and shortest diameters were recorded, and tumor volumes calculated using the formula: volume (mm³) = length × width² /2. Animals were euthanized if the tumor volume exceeded 1000 mm³ or if showed signs of distress. Four weeks following xenotransplant, tumors were excised.

### Viability assay

A total of 100 µL of transduced WERI-Rb1 cell suspension, containing 3000 cells per well, was plated in a 96-well plate and treated with 30 nM topotecan, which corresponds to the half-maximal inhibitory concentration (IC_50_) for non-target control cells (Supplementary Fig. 1A), or with vehicle (DMSO) for 96 hours. For Y79 cells, the IC_50_*ICIC*_50_ value for non-target control cells is established at 15 nM (Supplementary Fig. 1B). Subsequently, we added 10 µL of Cell Counting Kit-8 solution (Biosharp, BS350B) to each well of the plate. After incubating the plate for a period ranging from one to four hours in an incubator, absorbance was measured at 450 nm using a microplate reader. We normalized the intensity against DMSO-treated cells within each cell line before determining cell viability.

### Western blotting

Total proteins from cells and tissues were extracted using a RIPA lysis buffer kit (Beyotime Institute of Biotechnology). The protein concentration was quantified via a BCA assay (Beyotime Institute of Biotechnology). A total of 20 μg of protein from each sample was separated by electrophoresis on a 10% sodium dodecyl sulfate-polyacrylamide gel, followed by transfer to a polyvinylidene difluoride membrane. The membranes were blocked with 5% BSA for 2 h at room temperature and subsequently incubated overnight at 4 °C with primary antibodies. The following primary antibodies were utilized: Anti-GAPDH (1:10,000; cat. no. 10494-1-AP; ProteinTech Group, Inc.), anti-tubulin (1:1000; cat. no. sc-5274; Santa Cruz Biotechnology, Inc.), anti-Caspase-3 (1:500; cat. no. 9662; Cell Signaling Technology, Inc.), anti-MT-CYB (1:1000; ProteinTech Group, Inc.) and anti-ELF2 (1:500; ProteinTech Group, Inc.). Membranes were then treated with HRP-conjugated anti-rabbit IgG (1:10,000; cat. no. 7074 s; Cell Signaling Technology, Inc.) for 1 h at 37 °C before visualization using an enhanced chemiluminescence system (Thermo Fisher Scientific, Inc.). GAPDH or tubulin served as loading controls throughout the experiment. Band intensity was semi-quantified utilizing ImageJ software (version 1.51; National Institutes of Health).

### TUNEL assay

Retinoblastoma cells and 7 μm frozen sections derived from tumors were stained using the In Situ Cell Death Detection Kit, Red (Roche Diagnostics Corp., Indianapolis, IN, USA) following the manufacturer’s protocol. The samples were then counterstained with DAPI and examined under a fluorescent microscope (Leica Microsystems, Wetzlar, Germany). For in vitro experiments, at least 2000 cells per replicate (three replicates per group) were analyzed. For tumor tissues, five sections per tumor (covering the periphery to the center) and five random 20× fields per section were evaluated. The percentage of TUNEL-positive nuclei was quantified relative to the total DAPI-positive nuclei. Data in Fig. 2D and Fig. 6D were normalized to the mean value of the NT + NaCl group (set as 1.0), while data in other figures were presented as absolute percentages.

### RNA isolation and quantitative polymerase chain reaction (qPCR)

Total RNA was extracted from cells using TRIzol reagent (Invitrogen, CA, USA), and cDNA synthesis was conducted with a PrimeScript RT Reagent kit (Takara, Dalian, China) in accordance with the manufacturer’s instructions. Target mRNA levels were quantified by real-time PCR employing the SYBR Prime Script RT-PCR Kit on a LightCycler® 480 system (Roche, Switzerland), following the manufacturer’s protocol. Briefly, the reactions were incubated at 95 °C for 5 min, followed by 45 cycles of denaturation at 95 °C for 10 s, annealing at 60 °C for 10 s, and extension at 72 °C for 10 s. The relative mRNA levels were calculated using the 2−ΔΔCt method and normalized to human ACTB expression levels. Primer sequences are provided in Table S1.

### Cell cycle analysis by flow cytometry

Cell cycle distribution was analyzed using the Cell Cycle and Apoptosis Analysis Kit (Propidium Iodide (PI) Staining, MedChemExpress, HY-K1071) according to the manufacturer’s instructions. Briefly, cells were fixed in ice-cold 70% ethanol for at least 2 hours at 4°C. After washing with cold PBS, cells were resuspended and stained in the dark with 0.5 mL of PI/RNase A staining solution for 30 minutes at 37°C. Samples were then analyzed on a BD LSRFortessa™ X-20 flow cytometer using a 488 nm laser for excitation. The percentages of cells in the G0/G1, S, and G2/M phases were determined and analyzed using ModFit LT software (version 5.0.9, Verity Software House).

### Transcriptome sequencing

Transcriptome sequencing (RNA-seq) was conducted on transduced WERI-Rb1 cells treated with either vehicle or topotecan (30 nM for 96 h). The paired-end sequencing of cDNA libraries, utilizing a 2 × 150 bp configuration, was performed on an Illumina NovaSeq™ 6000 (LC-Bio Technology Co., Ltd., Hangzhou, China) in accordance with the manufacturer’s recommended protocol. FASTP software was employed to eliminate reads exhibiting adaptor contamination, low-quality bases, and undetermined bases using default parameters. Subsequently, sequence quality was further validated using the FASTP. StringTie was utilized to quantify mRNA expression levels by calculating FPKM (FPKM = [total_exon_fragments/mapped_reads(millions) × exon_length(kb)]). Differentially expressed mRNAs were identified based on a fold change greater than 2 or less than 0.5 and assessed through parametric F-tests comparing nested linear models (*p* value < 0.05), employing the R package edgeR.”

### Determination of mtDNA copy number

Genomic DNA was extracted from WERI-Rb1 and Y79 cells using the QIAamp DNA Mini Kit (QIAGEN, China). Relative mtDNA copy number was measured by a quantitative real-time PCR-based method. The primer sequences used were ND1-F, 5’-TGGCTCCTTTAACCTCTCCA-3’ and ND1-R, 5’-GGCGTATTCGATGTTGAAGC-3’ for mitochondrial NADH dehydrogenase 1, and HGB-F, 5’-GTGCACCTGACTCCTGAGGAGA-3’ and HGB-R, 5’-CCTTGATACCAACCTGCCCAG-3’, for Haemoglobin (nuclear DNA control), respectively. Real-time PCR (qRT-PCR) reactions were performed at 95 °C for 30 s followed by 35 cycles of 95 °C for 5 s and 60 °C for 30 s. The copy number was calculated based on threshold cycle values (Ct values) using the 2^−ΔΔCt^ method.

### ATP assays

The ATP content was determined using an Enhanced ATP Assay Kit (S0027; Beyotime Biotechnology, Shanghai, China), according to the manufacturer’s instructions. The concentration of ATP was calculated according to an ATP standard curve and expressed as nmol/OD730. Treatment with 1.25 μmol/L oligomycin inhibits the production of ATP via oxidative phosphorylation, allowing for the measurement of glycolytic ATP levels.

### Plasmids and transfection

The human ELF2 ORF clone expression plasmid (HG12537-UT) and pCMV3-untagged negative control vector (CV011; Sino Biological, Beijing, China) were transfected into WERI-Rb1 and Y79 cells separately using Lipofectamine™ 3000 transfection reagent (L3000075; Thermo Fisher Scientific, US). After 6 h of transfection, the medium was replaced with a fresh culture medium. Following 72 h of transfection, retinoblastoma cells were collected for further analysis.

### Immunohistochemistry

Four-micrometer-thick paraffin sections obtained from human tissues underwent standard Hematoxylin and Eosin (H&E) staining and immunohistochemistry (IHC) procedures following optimized protocols. Tissue specimens for IHC analysis were collected from a cohort comprising nine retinoblastoma patients who underwent treatment at the Zhongshan Ophthalmic Center. ELF2 expression was evaluated using a 1:50 dilution of anti-ELF2 antibody (Thermo Fisher Scientific, PA5-52247).

### Statistical analysis

No formal sample size calculation was performed; group sizes were determined based on feasibility and prior studies. The data are represented as the means ± standard deviation, and the differences between mean values were evaluated using GraphPad Prism 10 (GraphPad; San Diego, CA, USA) using Student’s *t* test for unpaired data, one-way ANOVA wherever appropriate, followed by Tukey’s or Bonferroni’s multiple comparisons tests. Homogeneity of variances between groups was assessed using the Brown-Forsythe test. The data were considered significantly different at a *P* value < 0.05. Tumor volume measurement and image quantifications were performed by investigators blinded to group allocation.

## Supplementary information

SUPPLEMENTAL MATERIAL

Table S1

SUPPLEMENTAL MATERIAL(WB)

## Data Availability

Raw RNA-seq data have been deposited in the Gene Expression Omnibus (GEO) under accession number GSE306615 (to be released upon publication). Processed counts and code are available on request.
