# Supplementary material for: Loss of ELF2 drives topotecan resistance in retinoblastoma revealed by genome-wide CRISPR-Cas9 screening
Source: Cell Death Dis. 2025 Dec 23;17(1):128. doi: 10.1038/s41419-025-08335-z (PMC12847836; doi:10.1038/s41419-025-08335-z)
Supplement: Supplementary file 1 — SUPPLEMENTAL MATERIAL [file 41419_2025_8335_MOESM1_ESM.docx]

**
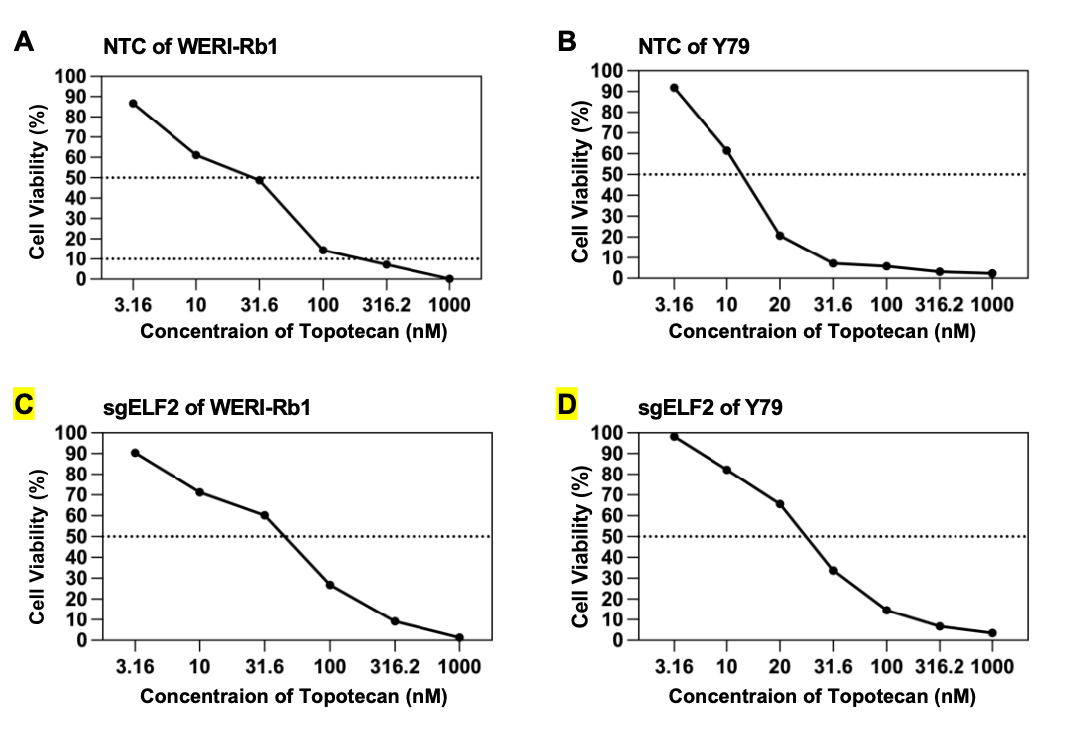
**

**Supplementary Figure 1.** The determination of topotecan concentration. **(A)** Cell viability of WERI-RB1 NTC cells under topotecan treatment for 96 hours. The IC50 of WERI-Rb1 NTC cells was ~30 nM. The IC90 of WERI-Rb1 NTC cells was ~100 nM. **(B)** Cell viability of Y79 NTC cells under topotecan treatment for 96 hours. The IC50 of Y79 NTC cells was ~15 nM.

**
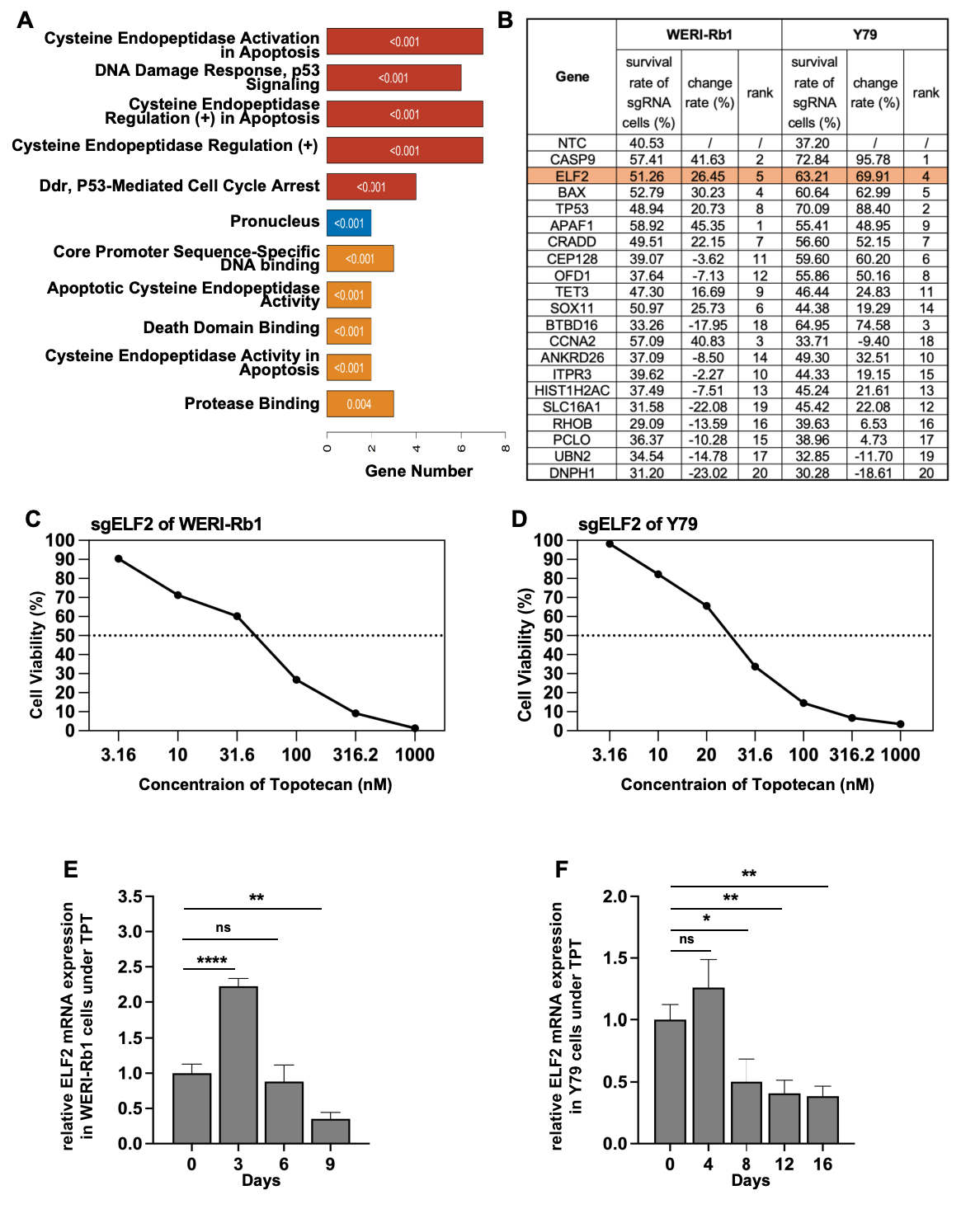
**

**Supplementary Figure 2.** Functional validation and expression analyses supporting the role of ELF2 in mediating topotecan resistance in retinoblastoma cells. **(A)** GO analysis of the top 50 topotecan positively selected hits in the CRISPR-Cas9 screen. **(B)** Survival rates of transduced WERI-Rb1 and Y79 cells were assessed. Under topotecan (TPT) treatment, survival rates for NTC cells were 40.53% in WERI-Rb1 and 37.20% in Y79 cells. Changes in survival rates compared to NTC cells were calculated using the formula: (S_(sgRNA+TPT)_/S_(sgRNA+veh)_-S_(NTC+TPT)_/S_(NTC+veh)_)/(S_(NTC+TPT)_/S_(NTC+veh)_). The change rates for sgELF2 cells ranked 5^th^ in WERI-Rb1 cells and 4^th^ in Y79 cells. **(C)** Cell viability of WERI-RB1 ELF2 knockout cells (sgELF2) under topotecan treatment for 96 hours. **(D)** Cell viability of Y79 ELF2 knockout cells (sgELF2) under topotecan treatment for 96 hours. (**E)** and **(F)** ELF2 mRNA expression in WERI-Rb1 and Y79 NTC cells under topotecan treatment (n=3). Statistical analysis was performed using one-way ANOVA and Tukey’s multiple comparison test (E, F); ****p < 0.0001, **p < 0.01, *p < 0.05.

**
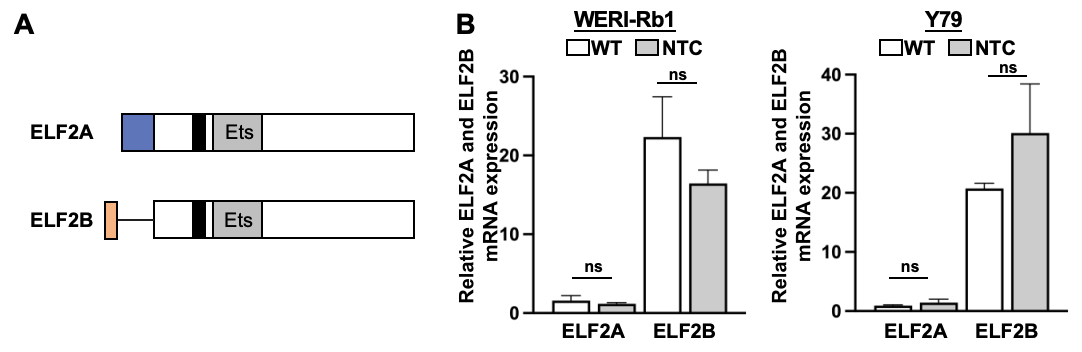
**

**Supplementary Figure 3.** Expression of ELF2 isoforms in retinoblastoma cells. **(A)** Schematic representation of ELF2 protein isoforms: shading indicates domains unique to ELF2A (blue) and ELF2B (orange), along with the common Ets DNA-binding domain (grey) and the putative bipartite nuclear localization signal (NLS) for ELF2A (amino acids 160-190) and ELF2B (amino acids 100-130) depicted in black. **(B)** Relative mRNA expression of ELF2A and ELF2B in WT and NTC cells of WERI-Rb1 and Y79.

**
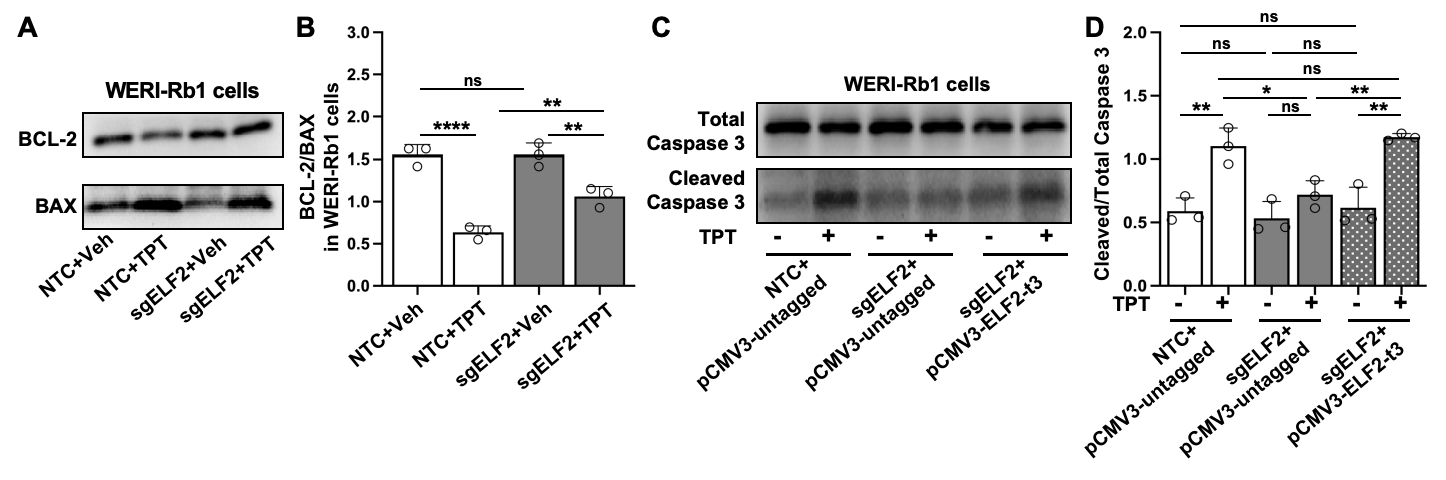
**

**Supplementary Figure 4.** ELF2 knockout alters apoptosis-related protein expression, while ELF2 re-expression restores caspase-3 activation in WERI-Rb1 cells following topotecan (TPT) treatment. **(A)** Representative western blot image of BCL-2 and BAX proteins in NTC or ELF2 knockout (sgELF2) WERI-Rb1 cells, with or without topotecan treatment. **(B)** Quantitative analysis of the BCL-2/BAX ratio (n=3). **(C)** Representative western blot image of total caspase-3 and cleaved caspase-3 proteins in NTC or ELF2 knockout (sgELF2) WERI-Rb1 cells transfected with either control vector (pCMV3-untagged) or ELF2 expression plasmid (pCMV3-ELF2-t3), with or without topotecan treatment. **(D)** Quantitative analysis of cleaved caspase-3 levels normalized to total caspase-3 (n=3). Data are presented as means ± SD. Statistical analysis was performed using one-way ANOVA and Tukey’s multiple comparison test; **p < 0.01, *p < 0.05.


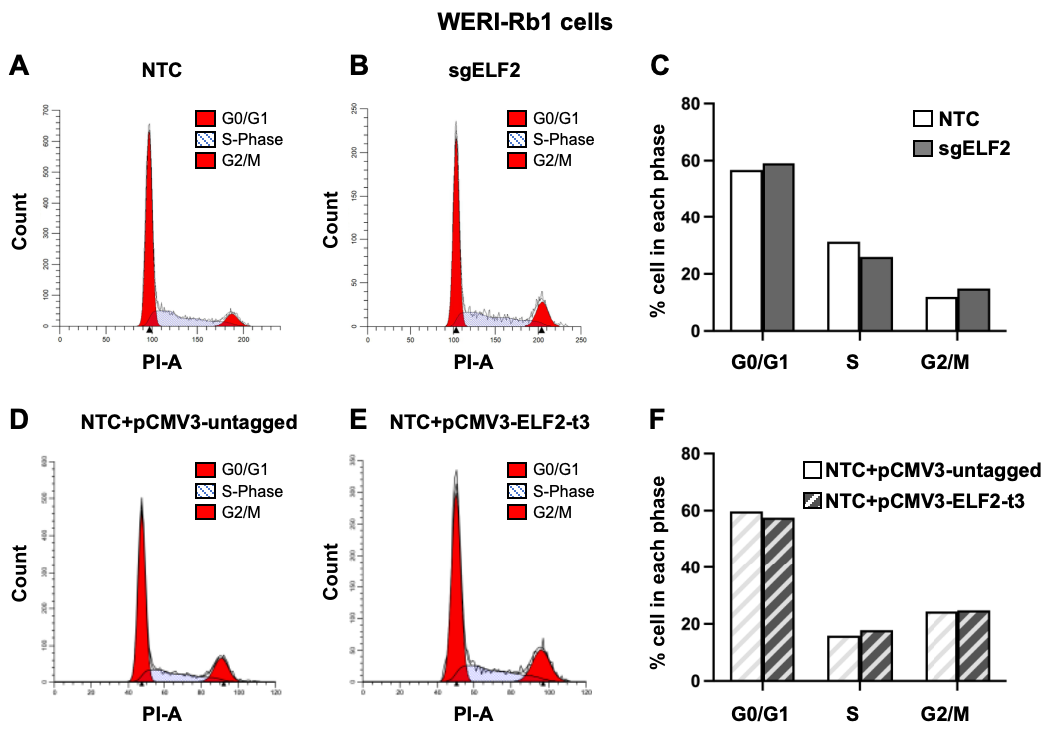


**Supplementary Figure 5.** ELF2 alteration does not affect cell cycle distribution in WERI-Rb1 cells. **(A)** Representative flow cytometry plots of cell cycle distribution in WERI-Rb1 NTC cells. **(B)** Representative plots of WERI-Rb1 sgELF2 cells. **(C)** Quantification of cell cycle phase distribution in WERI-Rb1 NTC and sgELF2 cells (n = 3). No significant differences in cell cycle were observed under topotecan-free conditions.


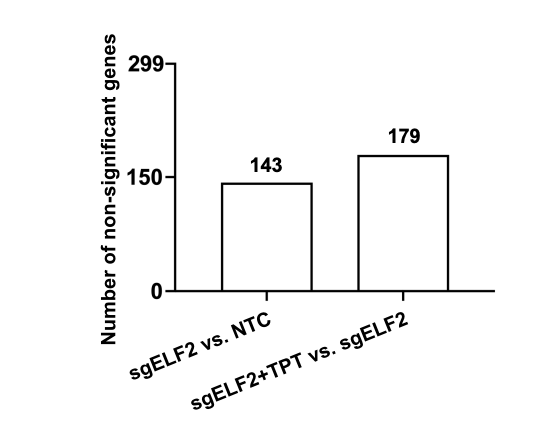


**Supplementary Figure 6.** Significant distribution of the 299 identified genes from 4C.

**
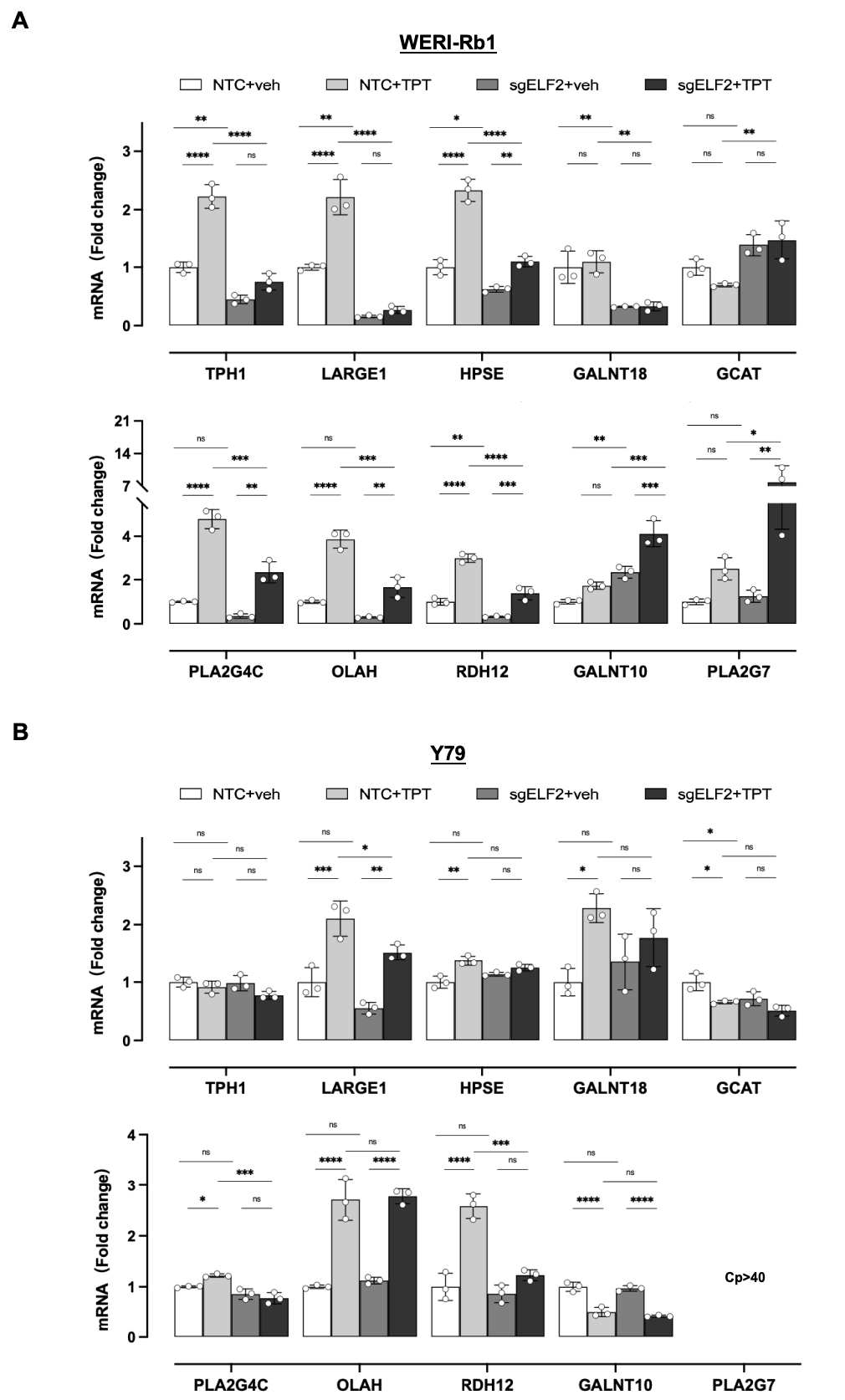
**

**Supplementary Figure 7.** The expression levels of genes associated with metabolic pathways are shown. **(A)** qPCR results of 10 candidate genes in WERI-Rb1 cells, identified from the metabolic pathways in Fig. 4E (genes with Cp>40 were excluded). **(B)** qPCR results of 10 candidate genes in Y79 cells, identified from the metabolic pathways in Fig. 4E (genes with Cp>40 were excluded). TPT: topotecan. Data are presented as means ± SD. Statistical analysis was performed using one-way ANOVA and Tukey’s multiple comparison test (A and B); ****p < 0.0001, ***p < 0.001, **p < 0.01, *p < 0.05.


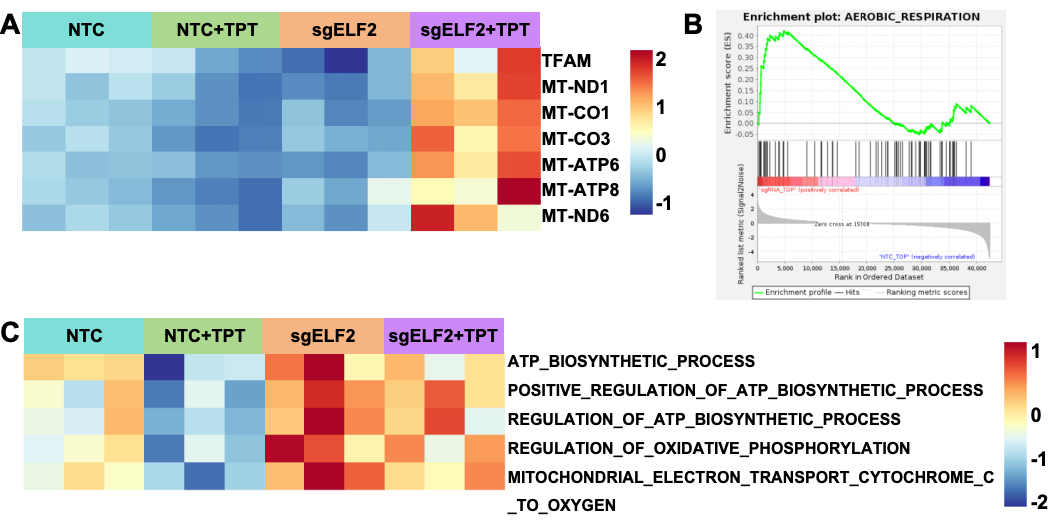


**Supplementary Figure 8.** Mitochondrial gene expression and pathway activity in response to ELF2 knockout and topotecan treatment. **(A)** qPCR validation of representative mitochondrial genes in the indicated experimental groups (n=3). **(B)** GSEA enrichment plots of AEROBIC_RESPIRATION pathways comparing sgELF2 + topotecan vs. NTC + topotecan. **(C)** GSVA heatmaps of mitochondrial and oxidative phosphorylation-related pathways across the four experimental groups.


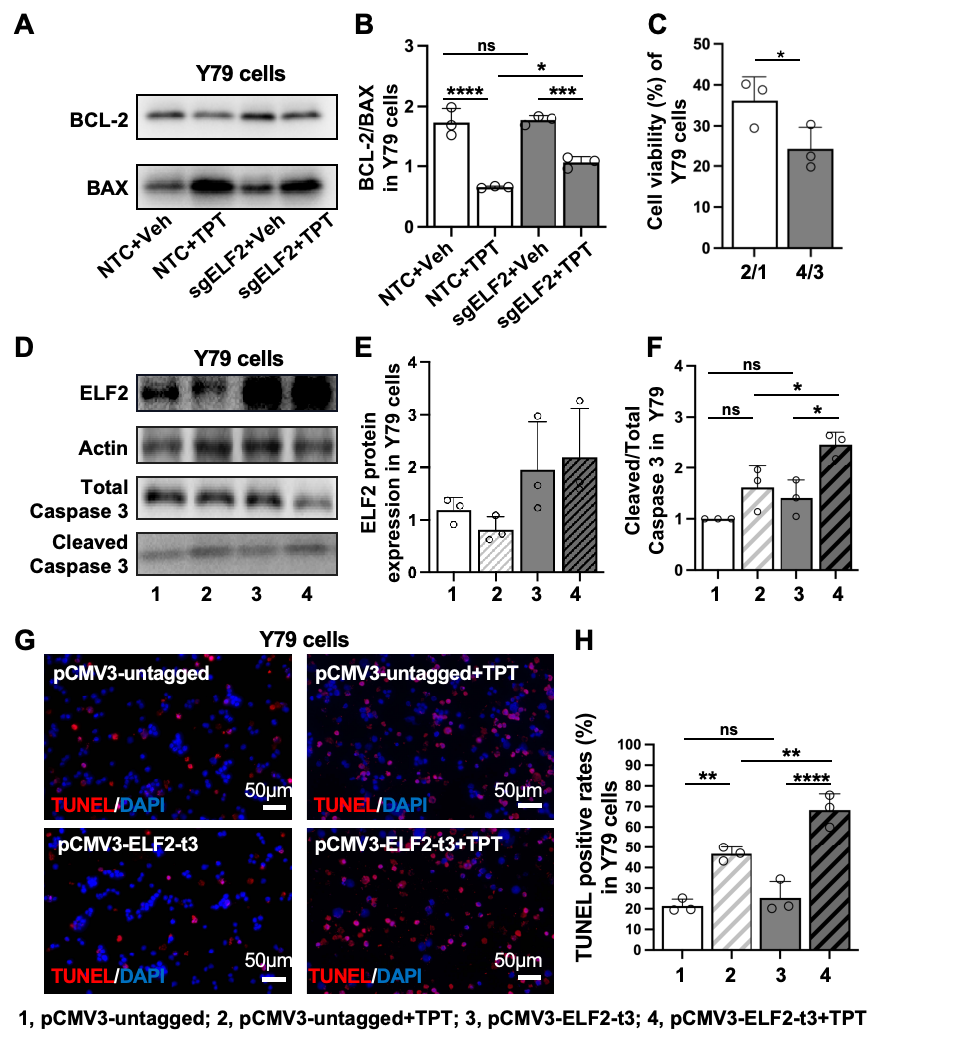


**Supplementary Figure 9.** ELF2 modulates apoptotic signaling and topotecan (TPT) sensitivity in Y79 cells. **(A)** Representative western blot image of BCL-2 and BAX proteins in NTC or ELF2 knockout (sgELF2) Y79 cells. **(B)** Quantitative analysis of the BCL-2/BAX ratio (n=3). (**C**) Relative cell viability of control cells (pCMV3-untagged) and ELF2 overexpressing cells (pCMV3-ELF2-t3) following treatment with 15 nM topotecan for 72 hours (n=3). The bars represent fold-change comparisons: 2/1 = (pCMV3-untagged + TPT) / (pCMV3-untagged), 4/3 = (pCMV3-ELF2-t3 + TPT) / (pCMV3-ELF2-t3). **(D)** Representative western blot image of ELF2 and caspase-3 proteins in ELF2 overexpressing (pCMV3-ELF2-t3) and control (pCMV3-untagged) Y79 cells. (**E** and **F**) Quantitative analysis of ELF2 protein, total caspase-3 and cleaved caspase-3 proteins (n=3). (**G** and **H**) Representative images and quantitative analysis of apoptotic cells by TUNEL assay in topotecan-treated ELF2 overexpressing (pCMV3-ELF2-t3) and control (pCMV3-untagged) cells (n=3; scale bar: 50 µm). Bar groups in panels E, F and H represent: 1 = pCMV3-untagged, 2 = pCMV3-untagged + TPT, 3 = pCMV3-ELF2-t3, 4 = pCMV3-ELF2-t3 + TPT. Data are presented as means ± SD. Statistical analysis was performed using two-tailed Student’s t-test (C) or one-way ANOVA and Tukey’s multiple comparison test (B, E, F and H); ****p < 0.0001, **p < 0.01, *p < 0.05.


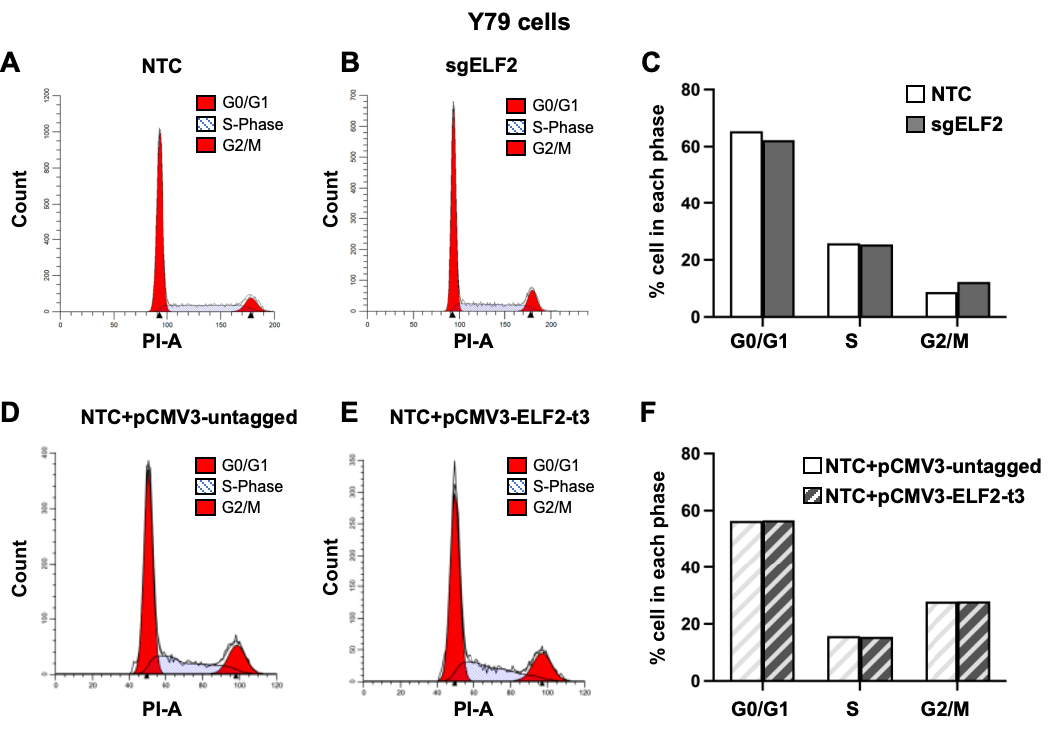


**Supplementary Figure 10.** ELF2 alteration does not affect cell cycle distribution in Y79 cells. **(A)** Representative flow cytometry plots of cell cycle distribution in Y79 NTC cells. **(B)** Representative plots of Y79 sgELF2 cells. **(C)** Quantification of cell cycle phase distribution in Y79 NTC and sgELF2 cells (n = 3). No significant differences in cell cycle were observed under topotecan-free conditions.


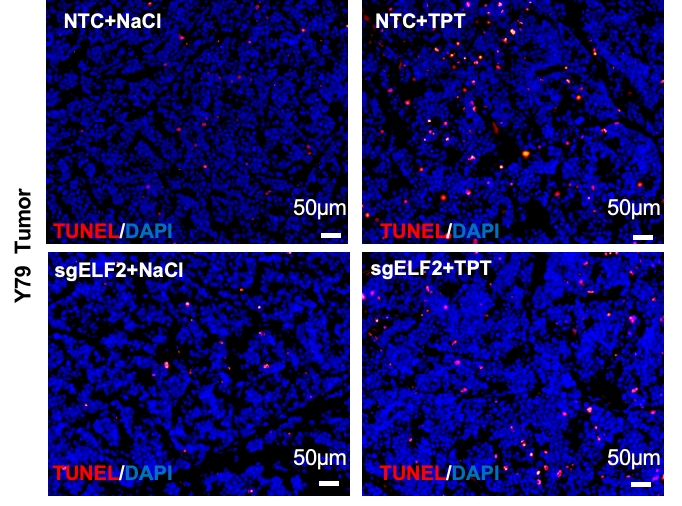


**Supplementary Figure 11.** Representative images (n = 5 for each group) of immunofluorescent staining for TUNEL-positive nuclei in Y79 tumors. The sgELF2+TPT group had fewer TUNEL-positive cells compared to the NTC+ topotecan (TPT) group. Scale bar: 50 μm.


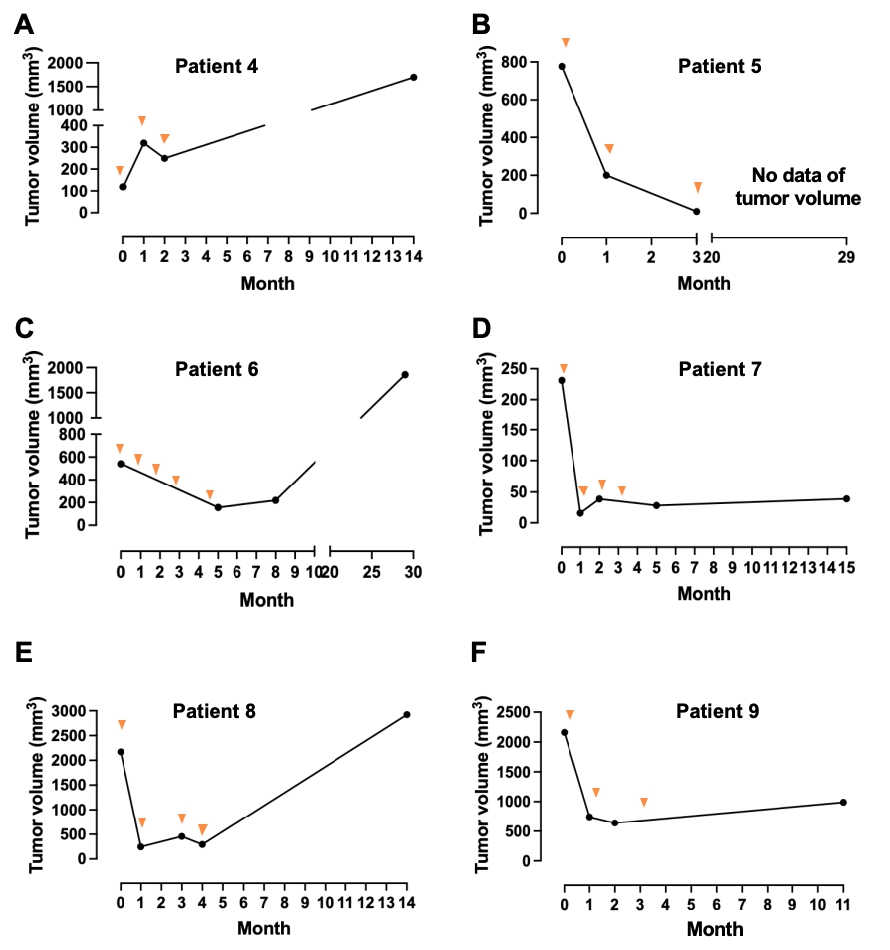


**Supplementary Figure 12.** Line graphs illustrating the tumor volumes of retinoblastoma patients. **(A)** Tumor growth in patient 4, whose retinoblastoma exhibited low to absent levels of ELF2 protein. (**B-F**) Tumor growth in patients 5-9, whose retinoblastomas exhibited high levels of ELF2 protein.
