# Supplementary material for: Loss of ELF2 drives topotecan resistance in retinoblastoma revealed by genome-wide CRISPR-Cas9 screening
Source: Cell Death Dis. 2025 Dec 23;17(1):128. doi: 10.1038/s41419-025-08335-z (PMC12847836; doi:10.1038/s41419-025-08335-z)
Supplement: Supplementary file 2 — Table S1 [file 41419_2025_8335_MOESM2_ESM.docx]

Table S1. List of Primers Used in This Study, Related to the Experimental Procedures

**Primers Used for Quantitative Real-Time PCR**

| Gene | Forward Primer | Reverse Primer |
| --- | --- | --- |
| MT-CYB | 5’CAATGGCGCCTCAATATTCT3’ | 5’CAGGAGGATAATGCCGATGT3’ |
| TPH1 | 5’ACGTCGAAAGTATTTTGCGGA3’ | 5’ACGGTTCCCCAGGTCTTAATC3’ |
| LARGE1 | 5’TGTCTGCGCCGGATACAATG3’ | 5’GGAGTCAGCAATAAGGTGGAAGT3’ |
| HPSE | 5’TCCTGCGTACCTGAGGTTTG3’ | 5’CCATTCCAACCGTAACTTCTCCT3’ |
| GALNT18 | 5’CCAGAGGTGAGCATCGTGTTC3’ | 5’GTTCCTCGTTACTGCTGTTGT3’ |
| GCAT | 5’CCTCAGCTCTGTCCGCTTTAT3’ | 5’GGATGCCGTCGATGATGGAG3’ |
| PLA2G4C | 5’TGCCGGAGTCTCATTTGTCC3’ | 5’GGGTGAACTCGAACCAGGTC3’ |
| OLAH | 5’GGAGACCAACCTAAGAGAACCA3’ | 5’AGCAAATCAGCTTAAAAGTTGCC3’ |
| RDH12 | 5’TGCTGCCAGTGAAATCCGAG3’ | 5’CATCATTACTCCCGCATTGTTGA3’ |
| GALNT10 | 5’TCAGCGCGTAGGAAATGGAG3’ | 5’GGAGAGAGCGATTCAAGGAGA3’ |
| PLA2G7 | 5’TCATCAGCATGGGTCAACAAAA3’ | 5’CCAAAGGGTGTCAAGGCGAT3’ |
| ND1 | 5’TGGCTCCTTTAACCTCTCCA3’ | 5’GGCGTATTCGATGTTGAAGC3’ |
| HGB | 5’GTGCACCTGACTCCTGAGGAGA3’ | 5’CCTTGATACCAACCTGCCCAG3’ |
