# Supplementary material for: Loss of ELF2 drives topotecan resistance in retinoblastoma revealed by genome-wide CRISPR-Cas9 screening
Source: Cell Death Dis. 2025 Dec 23;17(1):128. doi: 10.1038/s41419-025-08335-z (PMC12847836; doi:10.1038/s41419-025-08335-z)
Supplement: Supplementary file 3 — SUPPLEMENTAL MATERIAL(WB) [file 41419_2025_8335_MOESM3_ESM.pptx]

## Slide 1
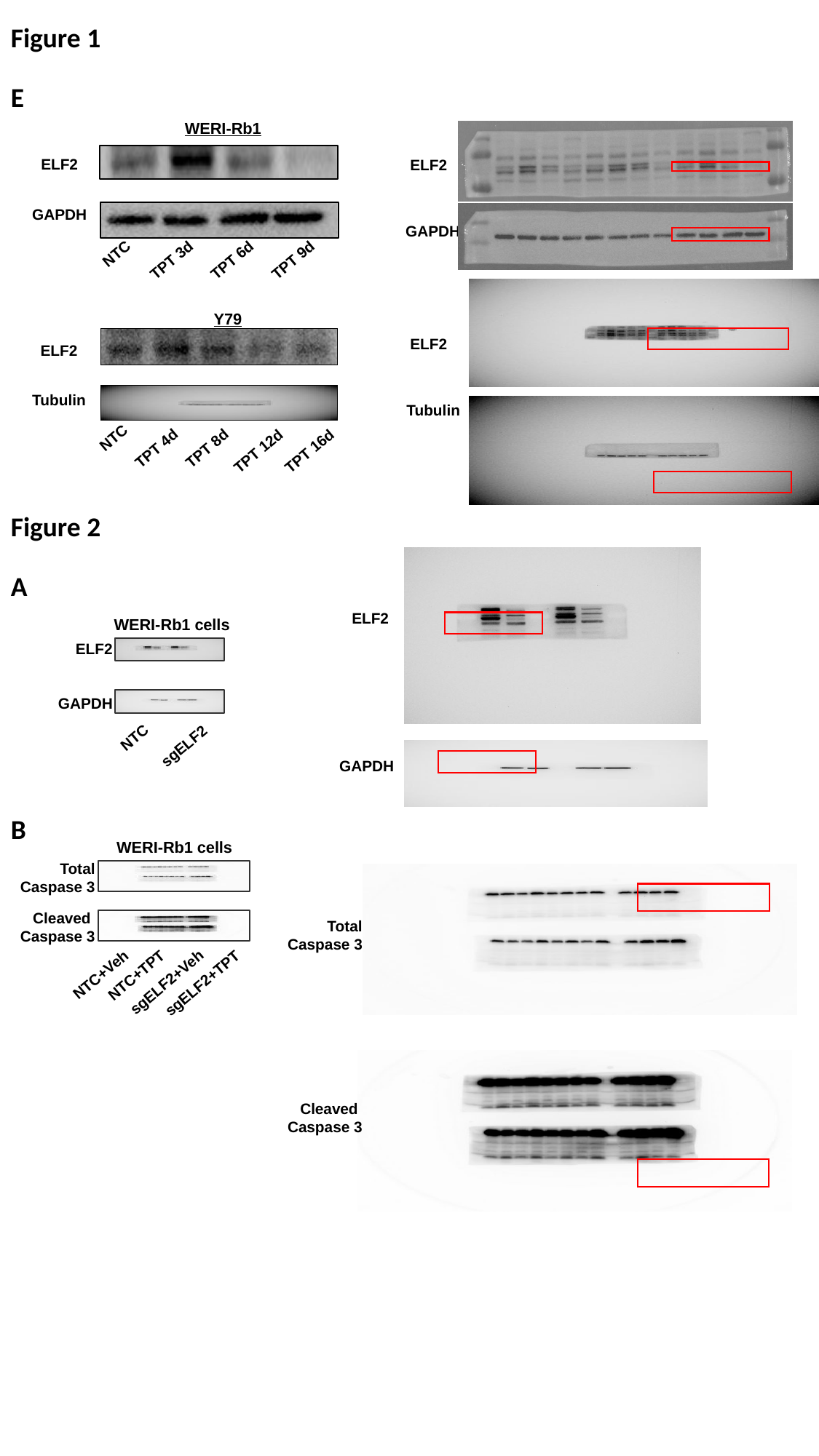

Figure 1
E
WERI-Rb1
ELF2
ELF2
GAPDH
GAPDH
NTC
 TPT 3d
TPT 6d
 TPT 9d
Y79
ELF2
ELF2
Tubulin
Tubulin
TPT 4d
TPT 8d
NTC
TPT 12d
TPT 16d
Figure 2
A
ELF2
WERI-Rb1 cells
ELF2
GAPDH
 NTC
sgELF2
GAPDH
B
WERI-Rb1 cells
Total
Caspase 3
Cleaved
Caspase 3
Total
Caspase 3
NTC+Veh
sgELF2+Veh
NTC+TPT
sgELF2+TPT
Cleaved
Caspase 3

## Slide 2
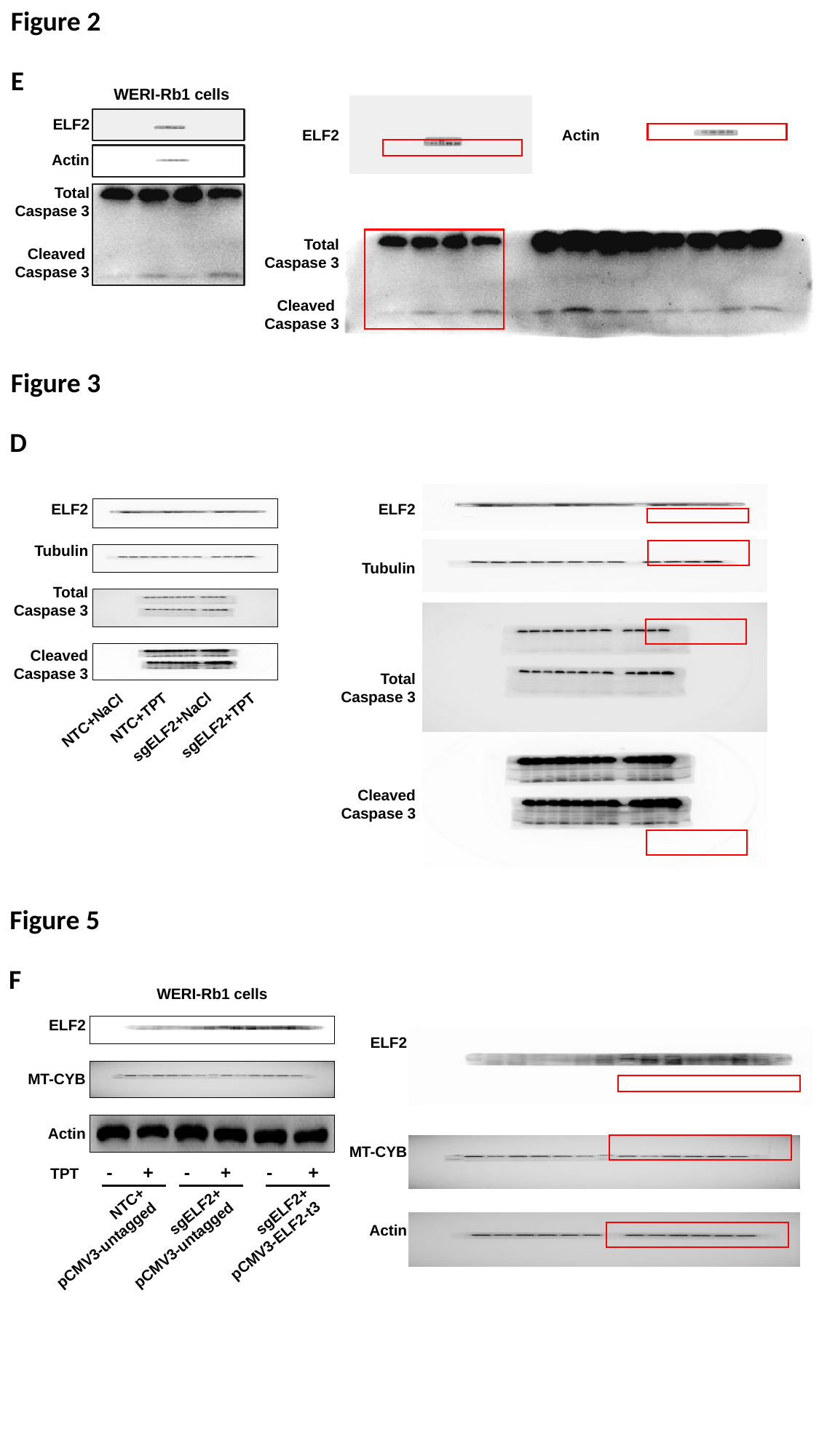

Figure 2
E
WERI-Rb1 cells
ELF2
ELF2
Actin
Actin
Total
Caspase 3
Total
Caspase 3
Cleaved
Caspase 3
Cleaved
Caspase 3
Figure 3
D
ELF2
ELF2
Tubulin
Tubulin
Total
Caspase 3
Cleaved
Caspase 3
Total
Caspase 3
NTC+TPT
NTC+NaCl
sgELF2+TPT
sgELF2+NaCl
Cleaved
Caspase 3
Figure 5
F
WERI-Rb1 cells
ELF2
ELF2
MT-CYB
Actin
MT-CYB
TPT - + - + - +
Actin
NTC+
pCMV3-untagged
sgELF2+
pCMV3-untagged
sgELF2+
pCMV3-ELF2-t3

## Slide 3
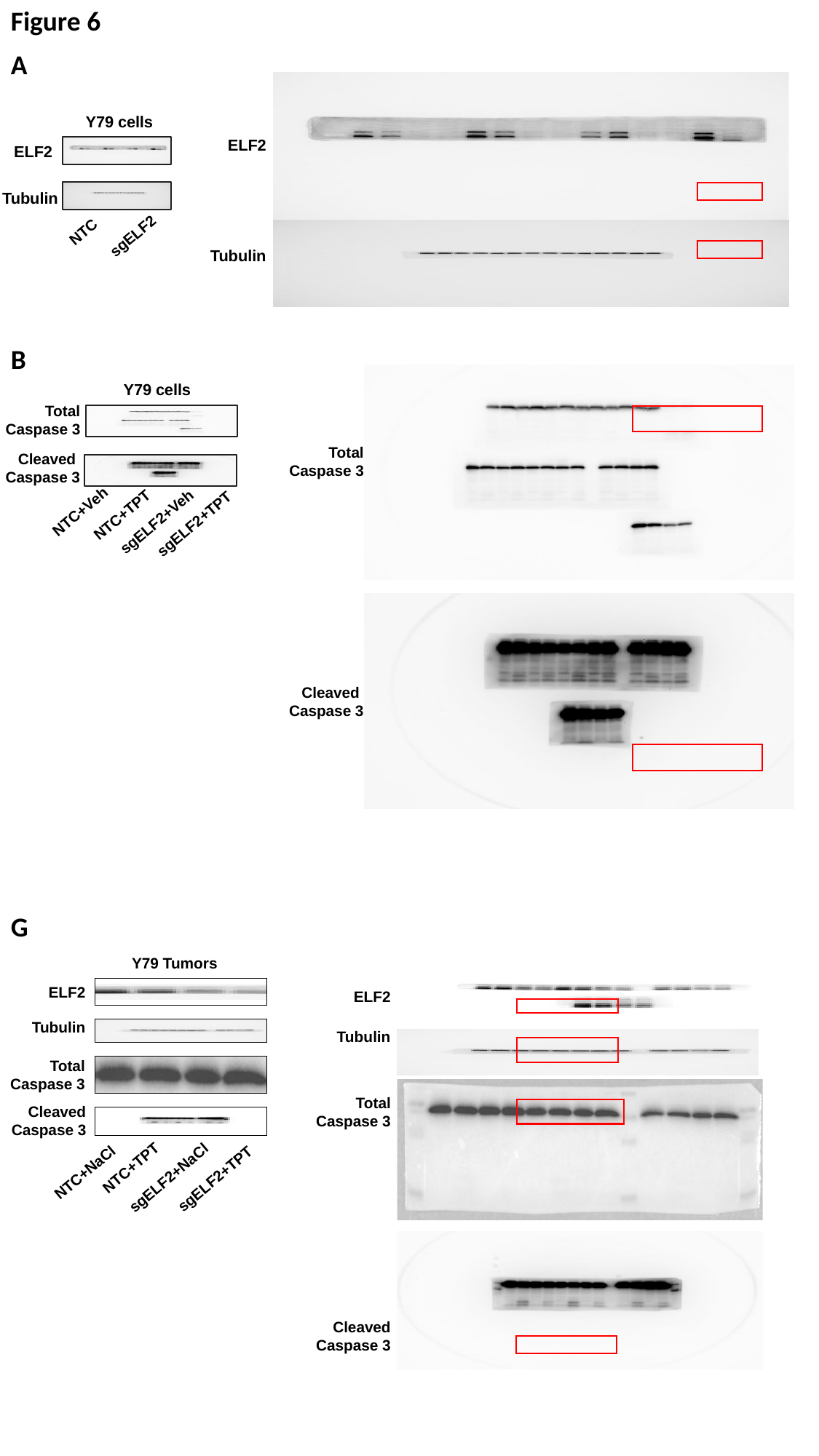

Figure 6
A
Y79 cells
ELF2
ELF2
Tubulin
 NTC
sgELF2
Tubulin
B
Y79 cells
Total
Caspase 3
Total
Caspase 3
Cleaved
Caspase 3
NTC+Veh
sgELF2+Veh
NTC+TPT
sgELF2+TPT
Cleaved
Caspase 3
G
Y79 Tumors
ELF2
ELF2
Tubulin
Tubulin
Total
Caspase 3
Total
Caspase 3
Cleaved
Caspase 3
NTC+TPT
NTC+NaCl
sgELF2+TPT
sgELF2+NaCl
Cleaved
Caspase 3
